# Concomitant Hepatorenal Dysfunction and Malnutrition in Valvular Heart Surgery: Long‐Term Prognostic Implications for Death and Heart Failure

**DOI:** 10.1161/JAHA.121.024060

**Published:** 2022-05-16

**Authors:** Yi‐Kei Tse, Chanchal Chandramouli, Hang‐Long Li, Si‐Yeung Yu, Mei‐Zhen Wu, Qing‐Wen Ren, Yan Chen, Pui‐Fai Wong, Ko‐Yung Sit, Daniel Tai‐Leung Chan, Cally Ka‐Lai Ho, Wing‐Kuk Au, Xin‐Li Li, Hung‐Fat Tse, Carolyn S. P. Lam, Kai‐Hang Yiu

**Affiliations:** ^1^ Division of Cardiology Department of Medicine The University of Hong Kong Shenzhen Hospital Shenzhen China; ^2^ Division of Cardiology Department of Medicine The University of Hong Kong Queen Mary Hospital Hong Kong China; ^3^ National Heart Centre Singapore Singapore; ^4^ Duke‐NUS Medical School Singapore; ^5^ Division of Cardiothoracic Surgery Department of Surgery The University of Hong Kong Queen Mary Hospital Hong Kong China; ^6^ Department of Cardiology Jiangsu Province Hospital and Nanjing Medical University First Affiliated Hospital Nanjing China; ^7^ University Medical Center Groningen Groningen Netherlands

**Keywords:** heart failure, hepatorenal dysfunction, malnutrition, risk‐stratification, valvular surgery, Valvular Heart Disease

## Abstract

**Background:**

Strategies to improve long‐term prediction of heart failure and death in valvular surgery are urgently needed because of an increasing number of procedures globally. This study sought to report the prevalence, changes, and prognostic implications of concomitant hepatorenal dysfunction and malnutrition in valvular surgery.

**Methods and Results:**

In 909 patients undergoing valvular surgery, 3 groups were defined based on hepatorenal function (the modified model for end‐stage liver disease excluding international normalized ratio score) and nutritional status (Controlling Nutritional Status score): normal hepatorenal function and nutrition (normal), hepatorenal dysfunction or malnutrition alone (mild), and concomitant hepatorenal dysfunction and malnutrition (severe). Overall, 32%, 46%, and 19% of patients were classified into normal, mild, and severe groups, respectively. Over a 4.1‐year median follow‐up, mild and severe groups incurred a higher risk of mortality (hazard ratio [HR], 3.17 [95% CI, 1.40–7.17] and HR, 9.30 [95% CI, 4.09–21.16], respectively), cardiovascular death (subdistribution HR, 3.29 [95% CI, 1.14–9.52] and subdistribution HR, 9.29 [95% CI, 3.09–27.99]), heart failure hospitalization (subdistribution HR, 2.11 [95% CI, 1.25–3.55] and subdistribution HR, 3.55 [95% CI, 2.04–6.16]), and adverse outcomes (HR, 2.11 [95% CI, 1.25–3.55] and HR, 3.55 [95% CI, 2.04–6.16]). Modified model for end‐stage liver disease excluding international normalized ratio and controlling nutritional status scores improved the predictive ability of European System for Cardiac Operative Risk Evaluation (area under the curve: 0.80 versus 0.73, *P*<0.001) and Society of Thoracic Surgeons score (area under the curve: 0.79 versus 0.72, *P*=0.004) for all‐cause mortality. One year following surgery (n=707), patients with persistent concomitant hepatorenal dysfunction and malnutrition (severe) experienced worse outcomes than those without.

**Conclusions:**

Concomitant hepatorenal dysfunction and malnutrition was frequent and strongly linked to heart failure and mortality in valvular surgery.

**Nonstandard Abbreviations and Acronyms**
CKD‐EPI: chronic kidney disease epidemiology collaboration
CONUT: controlling nutritional status
CVATS: Chinese Valvular Heart Disease Study
EuroSCORE II: European System for Cardiac Operative Risk Evaluation II
MELD‐XI: modified model for end‐stage liver disease excluding international normalized ratio
SHR: subdistribution hazard ratio
STS: Society of Thoracic Surgeons
TR: tricuspid regurgitation
VHD: valvular heart disease
ΔCONUT: 1‐year change in CONUT score
ΔMELD‐XI: 1‐year change in MELD‐XI score

**Clinical Perspective**

**What Is New?**

- In patients with valvular heart disease, hepatorenal dysfunction, represented by the modified model for end‐stage liver disease excluding international normalized ratio and nutritional status, by the Controlling Nutritional Status score, often coexist and are correlated with cardiac structure and function.
- Hepatorenal dysfunction and malnutrition are associated with an increased risk of heart failure and death after valvular surgery; beyond traditional cardiac surgery risk models, the modified model for end‐stage liver disease excluding international normalized ratio and Controlling Nutritional Status scores provide incremental value for risk stratification in valvular surgery.
- Deterioration of hepatorenal function and nutritional status, along with their persistent dysfunction 1 year after valvular surgery, confer poor long‐term prognosis.

**What Are the Clinical Implications?**

- Modified model for end‐stage liver disease excluding international normalized ratio and Controlling Nutritional Status scores, comprising simple and objective parameters obtained during routine assessment, could aid the prognostication of conventional risk‐scoring systems in valvular surgery.
- Pre‐ and postoperative clinical assessment can include an extracardiac workup to identify patients at high risk of adverse clinical outcomes.

Valvular interventions, either replacement or repair, remain the only definitive treatment to relieve symptoms and improve prognosis in patients with valvular heart disease (VHD).^1^ The immediate improvement in symptoms may, however, be counteracted by the development of heart failure (HF) late after valvular surgery, which portends a dismal prognosis frequently overlooked by traditional risk‐scoring models.^2^ Indeed, while the European System for Cardiac Operative Risk Evaluation (EuroSCORE) II and the Society of Thoracic Surgeons (STS) score are commonly used for risk stratification in valvular surgery,^3^, ^4^ they yield only modest discriminatory accuracy for predicting long‐term mortality and HF.^5^, ^6^ Furthermore, these scoring systems tend to misclassify risk, particularly among high‐risk individuals who are increasingly encountered in our aging population,^3^, ^5^, ^6^ along with an increasing prevalence of cardiovascular risk factors that drive the epidemic of VHD globally.^7^ As such, there is an urgent need to incorporate novel strategies to improve baseline risk stratification in patients with VHD.

Extracardiac manifestations, including end‐organ dysfunction^8^ and inflammation,^9^ are unique mechanistic factors that represent different expressions of VHD. In this context, hepatorenal function and nutritional status have emerged as key prognostic determinants in VHD,^10^, ^11^, ^12^ although current evidence^10^, ^12^, ^13^, ^14^ is limited to selected populations with short follow‐up, or insufficient adjustment of concomitant risk factors and medications. No study has reported the phenotype of concomitant hepatorenal dysfunction and malnutrition, leaving their consequences and changes following surgery unexplored. We sought to investigate the prevalence, risk factors, and prognostic role of hepatorenal function and nutritional status, as well as their incremental value to established risk‐stratification models in a large cohort of patients undergoing valvular surgery. Furthermore, we wanted to characterize the changes to hepatorenal function and nutritional status after valvular surgery to examine the role of longitudinal assessments in predicting adverse outcomes.

## Methods

### Data Availability

The data that support the findings of this study are available from the corresponding author upon reasonable request.

### Study Design and Population

This was a retrospective observational study that included 1080 consecutive patients with VHD undergoing valvular surgery at Queen Mary Hospital, Hong Kong between November 2012 and January 2021. Patients who presented for surgery primarily because of VHD were prospectively recruited into the Chinese Valvular Heart Disease Study database. From this cohort, patients with comprehensive laboratory assessment within the 3 months before valvular surgery and with at least 1 year of follow‐up were enrolled in the study. Patients with end‐stage liver (n=17) and renal disease (n=9) were excluded. End‐stage liver disease was defined by evidence of liver cirrhosis on abdominal imaging with episodes of ascites, variceal hemorrhage, or hepatic encephalopathy, while end‐stage renal disease was defined by an estimated glomerular filtration rate <15 mL/min per 1.73 m^2^ requiring hemodialysis. Baseline demographics between patients who were included (n=909) and excluded (n=171) for the present study were similar, except for a higher prevalence of atrial fibrillation, HF, and dyslipidemia among included patients (Table S1). This study was part of the Chinese Valvular Heart Disease Study to evaluate the pattern, pathophysiology, and clinical outcomes of VHD in Chinese patients.^15^ The study was approved by the Institutional Review Board of the West Cluster Hospital Authority of Hong Kong and written informed consents were obtained from all subjects.

### Clinical and Echocardiographic Parameters

Conventional cardiovascular risk factors (hypertension, diabetes, hyperlipidemia, atrial fibrillation, and smoking status), medical history (prior myocardial infarction and stroke), New York Heart Association functional class, comorbidities, and baseline medical therapy were reviewed based on electronic patient records and dispensing history at baseline visit. HF was diagnosed clinically based on signs and symptoms of volume overload with structural or functional abnormalities on transthoracic echocardiography. The cause of VHD was documented according to the predominant valvular lesion based on preoperative diagnosis and confirmed with surgical records. Left ventricular (LV) dimensions and systolic function, hemodynamics, and valvular lesion severity were assessed using an integrated approach based on M‐mode, 2‐dimensional and color, continuous‐ and pulsed‐wave Doppler echocardiography according to American Society of Echocardiography guidelines.^16^, ^17^, ^18^ Significant tricuspid regurgitation (TR) was defined as moderate or severe TR assessed using a multiparametric approach comprising qualitative, semiquantitative, and quantitative parameters.^18^

### Laboratory Measurements

Preoperative blood data represented the most recent laboratory analysis within the 3 months before valvular surgery. The estimated glomerular filtration rate was derived using the Chronic Kidney Disease Epidemiology Collaboration (CKD‐EPI) equation. To evaluate changes in hepatorenal function and nutrition after valvular surgery, postoperative blood data were also collected 1 year following surgery.

#### Assessment of Hepatorenal Function

Hepatorenal function was assessed using the modified model for end‐stage liver disease excluding international normalized ratio (MELD‐XI) score, which was calculated as 5.11×ln(serum total bilirubin)+11.76×ln(serum creatinine)+9.44. To avoid a negative score, 1.0 mg/dL was established as the minimum value of total bilirubin and creatinine. MELD‐XI score was selected as the principal measure of hepatorenal function because of its known association with clinical outcomes and its accurate reflection of hepatorenal function.^10^, ^11^, ^13^, ^14^

#### Assessment of Nutritional Status

Patients were screened for malnutrition using the Controlling Nutritional Status score (CONUT) that takes account of serum albumin, cholesterol, and total lymphocyte count. A score of 0 to 1 is deemed normal; scores of 2 to 4, 5 to 8, and 9 to 12 reflect mild, moderate, and severe malnutrition, respectively. The CONUT score was chosen because of its superior prognostic value and discrimination ability compared with other nutritional indices used in prior studies.^12^, ^19^

### End Points and Follow‐up

The end points of interest included all‐cause mortality, cardiovascular death, HF hospitalization, and major adverse cardiac events (defined as the composite of death and HF hospitalization). HF admission was defined as having symptoms or signs of HF and being prescribed diuretics during hospitalization. Information pertaining to HF hospitalization and death was ascertained from a detailed review of medical records, and follow‐up was complete for all patients.

### Statistical Analysis

Continuous data are presented as median with interquartile range and categorical data as frequencies and proportions. Differences among groups were tested using χ^2^ test for categorical variables and the Kruskal–Wallis H test for non‐normally distributed continuous variables. The correlation between MELD‐XI and CONUT scores was assessed by Spearman’s Rho. Linear and logistic regression were applied to identify predictors of hepatorenal dysfunction and malnutrition at baseline and 1‐year follow‐up. Receiver‐operating characteristic analysis was performed to determine the optimal cut‐off of MELD‐XI for predicting all‐cause mortality based on the Youden index. Using this cut‐off, patients were stratified according to the presence of hepatorenal dysfunction and malnutrition:

1. Normal: normal hepatorenal function and well‐nourished
2. Mild: hepatorenal dysfunction or malnutrition
3. Severe: hepatorenal dysfunction and malnutrition

Time‐to‐event data are summarized using Kaplan–Meier statistics, and log‐rank tests were used to compare survival across groups. Cox proportional hazards regression analyses were conducted to identify predictors of mortality and adverse events, and the proportional hazards assumptions were confirmed using Schoenfeld residuals. Variance inflation factors were used to determine whether significant collinearity was present between MELD‐XI and CONUT scores. To determine the relative prognostic importance of clinical covariates and risk scores, explainable log‐likelihood (χ^2^) was calculated for each predictor. The Grønnesby and Borgan test, likelihood ratio test, Akaike information criteria, and Bayesian information criteria were used to assess calibration of the adjusted models. The Fine‐Gray model was used to account for mutually exclusive end points; all‐cause mortality was considered as a competing risk for HF hospitalization and noncardiovascular death for cardiovascular death. The incremental prognostic value of hepatorenal function and nutritional status over traditional risk‐stratification models was assessed by multivariate stepwise block analysis. Formal risk‐reclassification analysis was performed by calculating increments in the Harrell *C*‐statistics, continuous net reclassification improvement, and integrated discrimination improvement. Changes in MELD‐XI (ΔMELD‐XI) and CONUT (ΔCONUT) scores were calculated as the difference between baseline and follow‐up scores.

All statistical analyses were performed using SPSS (Version 26.0, SPSS Inc) and R version 4.0.3. Statistical tests were 2‐sided, and a *P* value <0.05 denoted statistical significance.

## Results

### Patient Characteristics

All patients who met the study inclusion criteria formed the primary cohort (n=909). Patients with both pre‐ and postoperative laboratory indices of hepatorenal function and nutrition formed the secondary cohort (n=707) (Figure S1). Based on the optimal threshold of MELD‐XI score derived from receiver‐operating characteristic analysis (>12.43) and malnutrition defined by CONUT score (≥2), patients were stratified into 3 groups: 316 (35%) patients had normal hepatorenal function and were well‐nourished (normal); 416 (46%) had hepatorenal dysfunction or malnutrition (mild); and 177 (19%) had hepatorenal dysfunction and malnutrition (severe). Baseline characteristics according to hepatorenal function and nutrition phenotypes are presented in Table 1. During a median follow‐up of 4.1 years (interquartile range, 2.4 to 5 years), 101 (11%) patients died (54 [6%]) because of cardiovascular causes and 119 (13%) were hospitalized for HF.

**Table 1:** Baseline Characteristics of the Study Population According to Hepatorenal Function (MELD‐XI score) and Nutritional Status (CONUT score)

Characteristics	Overall (n=909)	Normal (Normal hepatorenal function and well‐nourished; n=316)	Mild (Hepatorenal dysfunction or malnutrition; n=416)	Severe (Hepatorenal dysfunction and malnutrition; n=177)	*P* value
Demographic and anthropometric characteristics					
Age, y	63 (57–69)	60 (54–65)^†,‡^	64 (57–71)^*^	65 (60–70)^*^	<0.001
Male	431 (47.4)	132 (41.8)^‡^	192 (46.2)^‡^	107 (60.5)^*,†^	<0.001
Height, cm	159 (153–166)	159 (154–165)	159 (153–166)	160 (153–166)	0.969
Weight, kg	58 (50–67)	61 (52–70)^†,‡^	57 (50–66)^*^	57 (49–67)^*^	0.002
Body mass index, kg/m^2^	23.0 (20.6–25.6)	23.8 (21.3–25.9)^†,‡^	22.7 (20.2–25.5)^*^	22.6 (19.7–25.4)^*^	0.002
NYHA Class III/IV	69 (7.6)	18 (5.7)^‡^	24 (5.8)^‡^	27 (15.3)^*,†^	<0.001
Cardiovascular risk factors and cardiovascular disease					
Hypertension	290 (31.9)	78 (24.7)^†,‡^	146 (35.1)^*^	66 (37.3)^*^	0.003
Diabetes	165 (18.2)	27 (8.5)^†,‡^	83 (20.0)^*,‡^	55 (31.1)^*,†^	<0.001
Dyslipidemia	231 (25.4)	64 (20.3)^†^	120 (28.8)^*^	47 (26.6)	0.028
Smoking	178 (19.6)	65 (20.6)	71 (17.1)	42 (23.7)	0.150
Prior myocardial infarction	37 (4.1)	6 (1.9)	21 (5.0)	10 (5.6)	0.507
Prior stroke	82 (9.1)	16 (5.1)^†,‡^	42 (10.2)^*^	4 (13.7)^*^	0.004
Heart failure	393 (43.2)	114 (36.1)^‡^	171 (41.1)^‡^	108 (61.0)^*,†^	<0.001
Atrial fibrillation	487 (53.6)	134 (42.4)^†,‡^	231 (55.5)^*,‡^	122 (68.9)^*,†^	<0.001
Comorbidities					
Chronic obstructive pulmonary disease	52 (5.7)	13 (4.1)	28 (6.7)	11 (6.2)	0.304
Cancer	52 (5.7)	19 (6.0)	26 (6.3)	7 (4.0)	0.525
Laboratory examination					
Hemoglobin, g/dL	12.9 (11.6–14.0)	13.3 (12.5–14.4)^†,‡^	12.8 (11.6–14.0)^*,‡^	11.3 (9.9–12.9)^*,†^	<0.001
White blood cell count, ×10^9^/L	5.9 (4.9–7.0)	6.0 (5.2–6.9)	5.9 (4.6–7.3)	5.7 (4.7–6.8)	0.287
Platelet count, ×10^9^/L	189 (157–227)	200 (174–234)^†,‡^	185 (153–222)^*,‡^	170 (128–213)^*,†^	<0.001
Creatinine, mg/dL	0.92 (0.78–1.14)	0.86 (0.76–1.00)^†,‡^	0.89 (0.77–1.06)^*,‡^	1.38 (1.12–1.61)^*,†^	<0.001
eGFR, mL/min per 1.73 m^2^	75.9 (60.9–90.1)	83.1 (72.8–95.1)^†,‡^	78.1 (65.7–90.7)^*,‡^	51.3 (36.9–61.5)^*,†^	<0.001
AST, U/L	27 (22–35)	26 (22–32)^†,‡^	27 (22–35)^*,‡^	30 (25–41)^*,†^	<0.001
ALT, U/L	21 (16–29)	22 (17–29)^‡^	21 (16–29)	20 (15–25)^*^	0.046
ALP, U/L	71 (58–90)	66 (55–83)^†,‡^	71 (58–87)^*,‡^	83 (64–124)^*,†^	<0.001
Total bilirubin, mg/dL	0.53 (0.75–1.14)	0.63 (0.47–0.84)^†,‡^	0.77 (0.56–1.08)^*,‡^	1.56 (0.80–2.20)^*,†^	<0.001
Total cholesterol, mg/dL	159 (134–188)	189 (167–211)^†,‡^	147 (128–169)^*,‡^	135 (116–154)^*,†^	<0.001
Albumin, g/dL	4.2 (4.0–4.4)	4.3 (4.1–4.5)^†,‡^	4.2 (4.0–4.4)^*,‡^	4.0 (3.7–4.3)^*,†^	<0.001
Valvular heart disease and echocardiographic variables					
MS ≥ moderate	229 (25.2)	81 (29.7)	109 (30.6)	39 (26.5)	0.657
MR ≥ moderate	411 (45.2)	149 (47.8)	170 (41.4)^‡^	92 (52.6)^†^	0.031
AS ≥ moderate	322 (35.4)	108 (38.3)	159 (41.7)	55 (34.6)	0.281
AR ≥ moderate	231 (25.4)	90 (30.3)	99 (24.5)	42 (24.1)	0.171
TR ≥ moderate	365 (40.2)	78 (24.7)^†,‡^	177 (42.5)^*,‡^	110 (62.5)^*,†^	<0.001
Chronic rheumatic heart disease	259 (28.5)	84 (26.6)	123 (29.6)	52 (29.4)	0.647
LV mass, g	224 (176–294)	221 (175–294)^†,‡^	219 (169–282)^*,‡^	249 (198–320)^*,†^	<0.001
LVEF, %	60 (55–60)	60 (55–60)^‡^	60 (55–60)^‡^	55 (50–60)^*,†^	<0.001
Preserved, ≥50%	763 (84.3)	278 (88.3)^‡^	348 (83.9)	137 (78.3)^*^	0.014
Midrange, 40%–49%	57 (6.3)	18 (5.7)	29 (7.0)	10 (5.7)	0.734
Reduced, <40%	55 (6.1)	11 (3.5)^‡^	24 (5.8)	20 (11.4)^*^	0.002
PASP, mm Hg	40 (35–50)	40 (30–45) ^†,‡^	40 (35–50)^*,‡^	45 (40–55) ^*,†^	<0.001
Medications					
ACEI	286 (31.5)	82 (25.9)^‡^	137 (32.9)	67 (37.9)^*^	0.016
ARB	142 (15.6)	45 (14.2)	72 (17.3)	25 (14.1)	0.437
Aldactone	117 (12.9)	20 (6.3)^†,‡^	51 (12.3)^*,‡^	46 (26.0)^*,†^	<0.001
β‐Blockers	374 (41.1)	116 (36.7)^‡^	171 (41.1)	87 (49.2)^*^	0.027
Calcium channel blockers	185 (20.4)	59 (18.7)	93 (22.4)	33 (18.6)	0.387
Digoxin	269 (29.6)	80 (25.3)^‡^	113 (27.2)^‡^	76 (42.9)^*,†^	<0.001
Statin	373 (41.0)	82 (25.9)^†,‡^	213 (51.2)^*^	78 (44.1)^*^	<0.001
Warfarin	417 (45.9)	115 (36.4)^†,‡^	198 (47.6)^*,‡^	104 (58.8)^*,†^	<0.001
Cardiac surgery risk‐stratification models					
EuroScore II	2.42 (1.33–4.50)	1.73 (1.01–2.85)^†,‡^	2.49 (1.53–4.72)^*,‡^	4.25 (2.77–8.82)^*,†^	<0.001
STS Score	1.49 (0.87–2.76)	1.12 (0.63–1.80)^†,‡^	1.63 (1.02–2.75)^*,‡^	3.02 (1.39–5.40)^*,†^	<0.001
Valvular surgery details					
Aortic valve replacement	460 (50.7)	164 (51.9)	214 (51.4)	82 (46.6)	0.482
Mitral valve procedure	554 (61.0)	192 (61.0)	245 (58.9)	117 (66.1)	0.258
Mitral valve replacement	295 (32.5)	97 (30.7)	138 (33.2)	60 (33.9)	0.701
Mitral valve repair	259 (28.6)	95 (30.2)	107 (25.7)	57 (32.6)	0.181
Tricuspid annuloplasty	319 (35.2)	77 (24.4)^†,‡^	151 (36.3)^*,‡^	91 (52.0)^*,†^	<0.001
Concomitant CABG	107 (11.8)	18 (5.7)^†,‡^	59 (14.2)^*^	30 (17.1)^*^	<0.001
Emergency operation	20 (2.2)	3 (0.9)	10 (2.4)	7 (4.0)	0.086
Inotropic support	198 (21.8)	55 (17.4)	100 (24.0)	43 (24.3)	0.065
Operative complications					
Major bleeding events	11 (1.2)	2 (0.6)	6 (1.4)	3 (1.7)	0.493
Stroke	4 (0.4)	3 (0.9)	0 (0.0)	1 (0.6)	0.152

Values are expressed as median (interquartile range) or number (percentage). *P* value by Kruskal–Wallis H test for non‐normally distributed continuous variables. *P* value by χ^2^ test for categorical variables (Bonferroni correction: *P<0.05 vs normal [normal hepatorenal function and well‐nourished]; ^†^ *P*<0.05 vs mild [hepatorenal dysfunction or malnutrition]; ^‡^ *P*<0.05 vs severe [hepatorenal dysfunction and malnutrition]). ACEI indicates angiotensin‐converting enzyme inhibitors; ALP, alkaline phosphatase; ALT, alanine aminotransaminase; AR, aortic regurgitation; ARB, angiotensin II receptor blockers; AS, aortic stenosis; AST, aspartate aminotransferase; CABG, coronary artery bypass grafting; CONUT, controlling nutritional status; eGFR, estimated glomerular filtration rate; EuroSCORE II, European System for Cardiac Operative Risk Evaluation II; LV, left ventricle; LVEF, left ventricular ejection fraction; MELD‐XI, modified model for end‐stage liver disease excluding international normalized ratio; PASP, pulmonary artery systolic pressure; MR, mitral regurgitation; MS, mitral stenosis; NYHA, New York Heart Association; STS score, Society of Thoracic Surgeons Predicted Risk of Mortality Score; and TR, tricuspid regurgitation.

### Clinical Associations, Prognostic Impact, and Discrimination Capacity of Combined Hepatorenal Dysfunction and Malnutrition

Multiple markers of hepatic function (aspartate aminotransferase, alkaline phosphatase, total bilirubin, and albumin), renal function (estimated glomerular filtration rate), together with echocardiographic parameters of the left heart (left ventricular ejection fraction and LV mass), pulmonary artery systolic pressure, and TR became progressively abnormal from normal to severe hepatorenal dysfunction and malnutrition. Correlates of concomitant hepatorenal dysfunction and malnutrition (severe) were EuroSCORE II, STS score, LV mass, significant TR, and pulmonary artery systolic pressure (Table S2A). MELD‐XI scores were modestly correlated with CONUT scores (R=0.36, *P*<0.001) without significant collinearity (mean variance inflation factor =1.17) (Figure S2).

**Figure 1:**
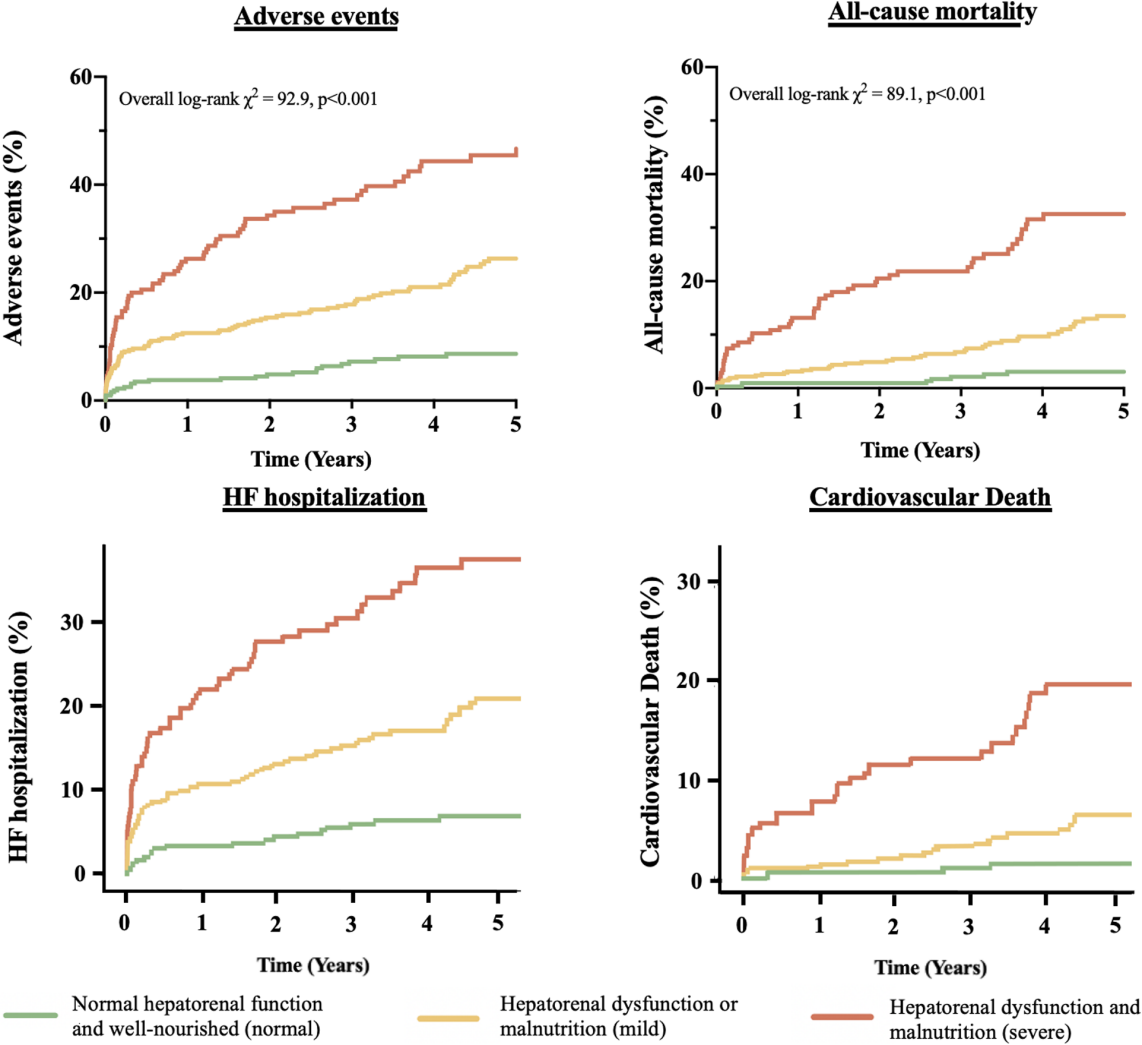
Cumulative incidence curves for all‐cause mortality, adverse events, heart failure hospitalization, and cardiovascular death by baseline hepatorenal dysfunction (MELD‐XI) and malnutrition (CONUT). CONUT indicates Controlling Nutritional Status score; and MELD‐XI, Model for End‐Stage Liver Disease excluding international normalized ratio.

A graded increase in mortality, cardiovascular death, HF hospitalization, and adverse outcomes was observed in patients from normal to severe groups, with a clear step‐up in event risk for patients in the severe group (Figure 1; Tables S2B through S2D). This association remained consistent in fully adjusted models (Table 2) regardless of the type of valvular surgery performed (aortic valve replacement and mitral valve surgery; Tables S3A and S3B) and the cause of VHD (chronic rheumatic heart disease and non–chronic rheumatic heart disease; Tables S3C and S3D). Landmark analysis excluding 30‐day mortality also yielded similar results (Table S4). Within the mild group, there were no significant differences in survival (92.3% versus 89.7%, *P*=0.530) and adverse outcomes (79.5% versus 78.0%, *P*=0.773) between the subgroup with hepatorenal dysfunction (n=39) and malnutrition alone (n=377) (Figure S3). The prevalence, clinical correlates, and prognostic implications of hepatorenal dysfunction and malnutrition alone are illustrated in Tables S5A through S6E. The MELD‐XI score was the most important predictor of mortality while the CONUT score was the strongest predictor of adverse events (Figure S4). The adjusted models demonstrated good calibration for predicting both all‐cause mortality and adverse events (Table S7).

**Table 2:** Cox Proportional Hazards Analyses of Baseline Hepatorenal function (MELD‐XI) and Nutritional Status (CONUT) for Predicting All‐Cause Mortality

	Overall population					
	Univariate analysis		Multivariate analysis (EuroSCORE II model)		Multivariate analysis (STS score model)	
	HR (95% CI)	*P* value	HR (95% CI)	*P* value	HR (95% CI)	*P* value
Demographic and anthropometric characteristics						
Age, y	1.06 (1.04–1.08)	<0.001				
Male	1.09 (0.74–1.61)	0.667				
Body mass index, kg/m^2^	1.02 (0.97–1.08)	0.398				
NYHA Class III/IV	1.73 (0.96–3.09)	0.067				
Cardiovascular risk factors and cardiovascular disease						
Hypertension	2.01 (1.36–2.97)	<0.001	1.82 (1.13–2.93)	0.014		
Diabetes	3.16 (2.12–4.70)	<0.001				
Smoking	0.86 (0.52–1.44)	0.575				
Dyslipidemia	2.05 (1.38–3.06)	<0.001	1.62 (1.02–2.57)	0.042	1.79 (1.15–2.78)	0.010
Prior myocardial infarction	2.03 (0.98–4.17)	0.055				
Prior stroke	2.32 (1.39–3.86)	0.001	1.61 (0.93–2.78)	0.088	1.70 (0.95–3.06)	0.074
Atrial fibrillation	1.33 (0.89–1.98)	0.159				
Heart failure	1.47 (1.00–2.18)	0.052				
Laboratory examination						
Hemoglobin, g/dL	0.66 (0.60–0.73)	<0.001				
eGFR, mL/min per 1.73 m^2^	0.97 (0.96–0.98)	<0.001				
Total bilirubin, mg/dL	1.42 (1.15–1.74)	<0.001				
Total cholesterol, mg/dL	0.984 (0.978–0.990)	<0.001				
Albumin, mg/dL	0.38 (0.24–0.60)	<0.001				
Valvular heart disease and echocardiographic variables						
MS ≥ moderate	0.86 (0.52–1.43)	0.566				
MR ≥ moderate	0.46 (0.30–0.71)	<0.001	0.50 (0.29–0.88)	0.015		
AS ≥ moderate	1.42 (0.94–2.15)	0.1				
AR ≥ moderate	0.91 (0.58–1.44)	0.701				
TR ≥ moderate	2.28 (1.53–3.39)	<0.001	2.38 (1.40–4.04)	0.002		
Chronic Rheumatic Heart Disease	1.35 (0.89–2.04)	0.157				
LV mass, g	1.001 (0.999–1.003)	0.221				
LVEF, %	0.98 (0.96–1.00)	0.019				
PASP, mm Hg	1.01 (0.99–1.02)	0.370				
Medications						
ACEI	1.48 (0.99–2.20)	0.056				
ARB	0.96 (0.55–1.66)	0.876				
β‐Blockers	0.79 (0.53–1.19)	0.259				
Calcium channel blockers	1.04 (0.64–1.68)	0.876				
Digoxin	1.44 (0.97–2.16)	0.074				
Statin	1.44 (0.97–2.13)	0.067				
Warfarin	1.49 (1.00–2.20)	0.048	1.15 (0.68–1.95)	0.600	1.47 (0.91–2.36)	0.113
Valvular surgery details						
Aortic valve replacement	1.63 (1.09–2.44)	0.017	1.30 (0.81–2.10)	0.281	1.36 (0.77–2.41)	0.288
Mitral valve procedure	0.56 (0.38–0.83)	0.004	0.67 (0.39–1.15)	0.149	0.47 (0.27–0.84)	0.010
Mitral valve replacement	0.75 (0.48–1.17)	0.198				
Mitral valve repair	0.64 (0.39–1.03)	0.065				
Tricuspid annuloplasty	1.36 (0.92–2.02)	0.126				
Concomitant CABG	1.28 (0.74–2.22)	0.376				
Cardiac surgery risk‐stratification models						
EuroScore II	1.06 (1.05–1.08)	<0.001	1.03 (1.01–1.06)	0.006		
STS Score	1.28 (1.20–1.36)	<0.001			1.16 (1.07–1.25)	<0.001
Combined evaluation of hepatorenal function and nutritional status						
Normal (Normal hepatorenal function and well–nourished)	1.00		1.00		1.00	
Mild (Hepatorenal dysfunction or malnutrition)	4.28 (2.01–9.11)	<0.001	3.17 (1.40–7.17)	0.006	2.93 (1.35–6.38)	0.007
Severe (Hepatorenal dysfunction and malnutrition)	13.86 (6.58–29.23)	<0.001	9.30 (4.09–21.16)	<0.001	8.07 (3.63–17.95)	<0.001

ACEI indicates angiotensin‐converting enzyme inhibitors; AR, aortic regurgitation; ARB, angiotensin II receptor blockers; AS, aortic stenosis; CABG, coronary artery bypass grafting; CONUT, controlling nutritional status; eGFR, estimated glomerular filtration rate; EuroSCORE II, European System for Cardiac Operative Risk Evaluation II; HR, hazard ratio; LV, left ventricle; LVEF, left ventricular ejection fraction; MELD‐XI, modified model for end‐stage liver disease excluding international normalized ratio; MR, mitral regurgitation; MS, mitral stenosis; NYHA, New York Heart Association; PASP, pulmonary artery systolic pressure; STS score, Society of Thoracic Surgeons Predicted Risk of Mortality Score; and TR, tricuspid regurgitation.

**Figure 2:**
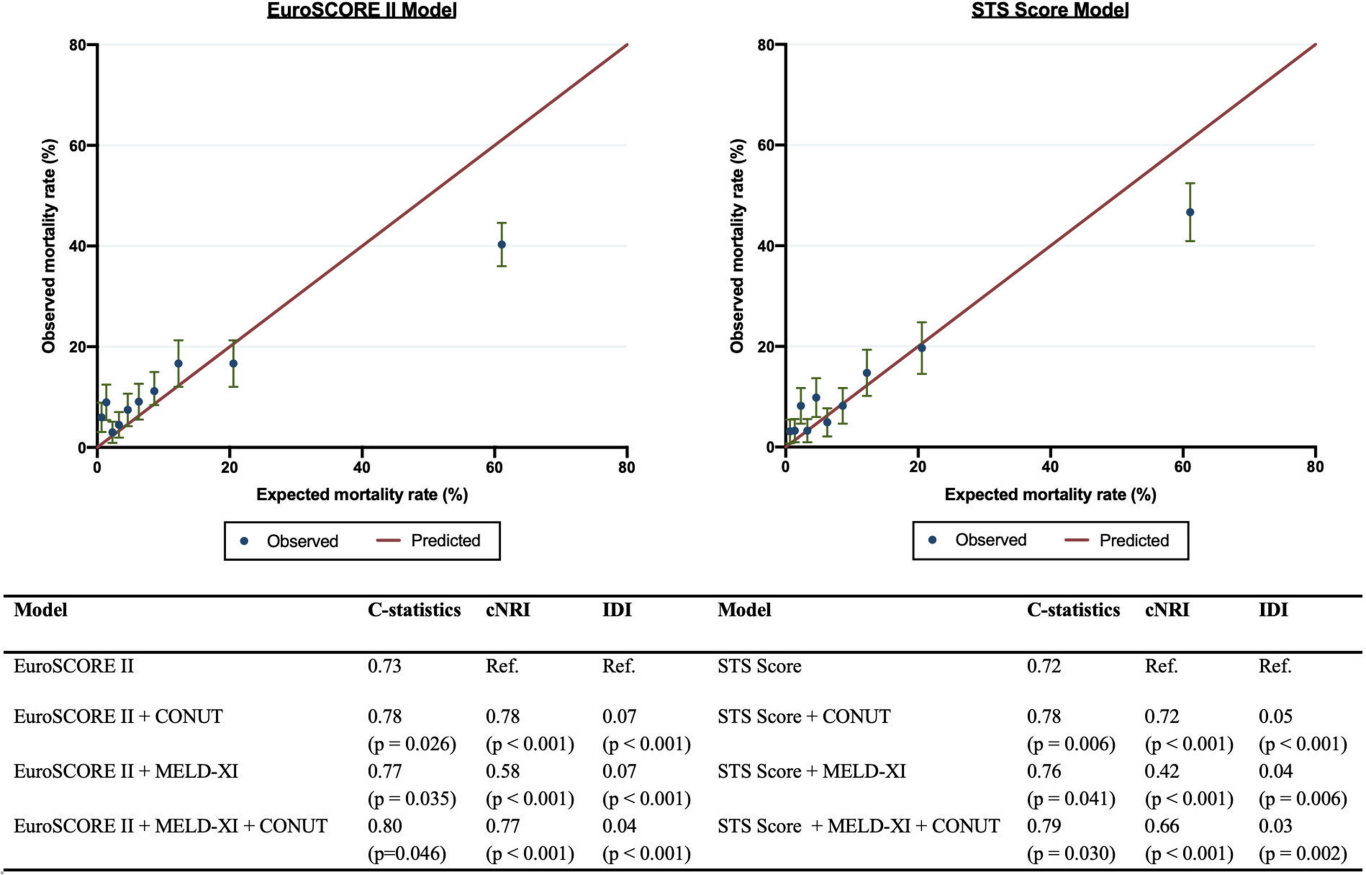
Discrimination and calibration of MELD‐XI and CONUT scores when added to EuroSCORE II and STS score for predicting all‐cause mortality. cNRI indicates continuous net reclassification improvement; CONUT, Controlling Nutritional Status score; EuroSCORE II, European System for Cardiac Operative Risk Evaluation II; IDI, integrated discrimination improvement; MELD‐XI, Model for End‐Stage Liver Disease excluding international normalized ratio; and STS score, Society of Thoracic Surgeons Predicted Risk of Mortality Score.

The addition of the MELD‐XI score provided incremental prognostic value (χ^2^ increased by 69.5, *P*<0.001) and discrimination improvement by *C*‐statistic (0.77 versus 0.73, *P*=0.035) over EuroSCORE II (Figure 2). Likewise, the addition of the CONUT score to EuroSCORE II significantly improved model fit (χ^2^ increased by 48.7, *P*<0.001) and discrimination (*C*‐statistic 0.78 versus 0.73, *P*=0.026). The continuous net reclassification improvement and integrated discrimination improvement for all‐cause mortality also increased after adding MELD‐XI (0.58 and 0.07, *P*<0.001) and CONUT score (0.78 and 0.07, *P*<0.001) to EuroSCORE II. Notably, adding CONUT to a combined model of EuroSCORE II and MELD‐XI or MELD‐XI to EuroSCORE II and CONUT provided incremental prognostic value (χ^2^ increased by 48.1 and 68.9, respectively, *P*<0.001 for both) and significant reclassification improvement (0.77 [95% CI, 0.57–0.96]; 0.45 [95% CI, 0.25–0.66], *P*<0.001 for both). Similar results were observed when STS score replaced EuroSCORE II in the model (Figure 2). Sensitivity analysis replacing the CONUT score with serum albumin did not yield similar incremental value, although significant reclassification improvement was seen in the STS score model (Table S8). The inclusion of MELD‐XI and CONUT scores improved calibration of the EuroSCORE II and STS score models (Tables S9A and S9B).

### Dynamic Changes of Hepatorenal Function and Nutrition at 1‐Year Follow‐up

At 1 year after surgery, 707 patients underwent laboratory examination (median interval, 14.5 months [interquartile range, 13–18 months]). The primary and secondary cohorts had similar clinical characteristics (Table S10).

At 1‐year follow‐up post valvular surgery, 15% of patients had concomitant hepatorenal dysfunction and malnutrition (severe), 41% of patients had either condition (mild), and 43% had neither condition (normal hepatorenal function and nutrition). While hepatorenal function and nutrition deteriorated in 105 (15%) patients after valvular surgery, they remained static in 454 (64%) and had improved in 148 (21%). ΔMELD‐XI was correlated with New York Heart Association class III/IV and ΔCONUT score (Table S11A) and was significantly associated with mortality (HR, 1.09 [95% CI, 1.02–1.18], *P*=0.020), cardiovascular death (SHR, 1.14 [95% CI, 1.05–1.24], *P*=0.003), HF hospitalization (SHR, 1.10 [95% CI, 1.03–1.17], *P*=0.003), and adverse events (HR, 1.08 [95% CI, 1.03–1.14], *P*=0.001). Similarly, ΔCONUT was associated with EuroSCORE II and ΔMELD‐XI (Table S11B) and was a significant predictor of mortality (HR, 1.20 [95% CI, 1.01–1.43], *P*=0.040), HF hospitalization (SHR, 1.23 [95% CI, 1.06–1.42], *P*=0.006), and adverse events (HR, 1.18 [95% CI, 1.02–1.35], *P*=0.020).

**Figure 3:**
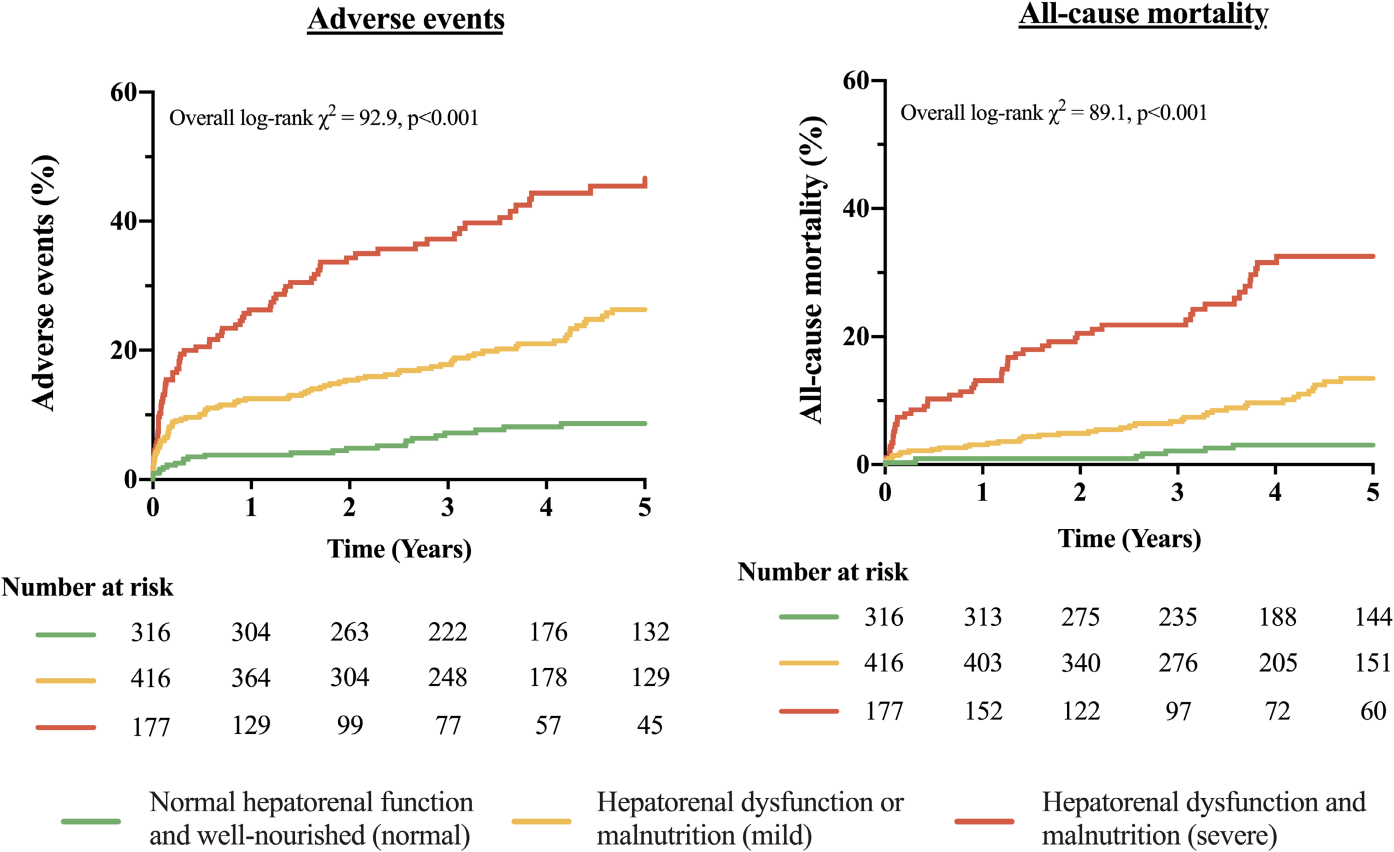
Kaplan–Meier curves for all‐cause mortality and adverse events by postoperative hepatorenal dysfunction (MELD‐XI) and malnutrition (CONUT). CONUT indicates Controlling Nutritional Status score; and MELD‐XI, Model for End‐Stage Liver Disease excluding international normalized ratio.

Concomitant hepatorenal dysfunction and malnutrition (severe) was evident in 108 (15%) patients following surgery, of whom it was newly developed in 44 (41%). Risk factors for their presence were LV mass, significant TR, EuroSCORE II, and baseline MELD‐XI and CONUT scores (Table S11C). Patients with persistent hepatorenal dysfunction and malnutrition following valvular surgery had worse survival (log‐rank χ^2^ 65.2, *P*<0.001) and adverse outcomes (log‐rank χ^2^ 90.4, *P*<0.001) compared with those with preserved hepatorenal function and/or nutrition (Figure 3). Notably, concomitant hepatorenal dysfunction and malnutrition (severe) exhibited a higher risk of mortality (HR, 4.35 [95% CI, [1.91–9.94], *P*<0.001), cardiovascular death (SHR, 10.74 [95% CI, 3.51–32.81], *P*<0.001), HF hospitalization (SHR,5.27 [95% CI, 3.02–9.19], *P*<0.001), and adverse events (HR, 3.61 [95% CI, 1.99–6.55], *P*<0.001).

## Discussion

Based on a comprehensive analysis of clinical, echocardiographic, and laboratory data of a large cohort of patients undergoing valvular surgery, we report the following results: (1) hepatorenal dysfunction and malnutrition were prevalent and often coexisted in VHD; (2) hepatorenal dysfunction and malnutrition, correlated with echocardiographic indices of cardiac structure and function, were associated with excess HF and mortality, with a greater step‐up in event risk for concomitant hepatorenal dysfunction and malnutrition (severe) than for either phenotype alone; (3) MELD‐XI and CONUT scores provided independent and incremental value for risk‐stratification over EuroSCORE II and STS score; and (4) deterioration of hepatorenal function (ΔMELD‐XI) and nutritional status (ΔCONUT), along with their persistent dysfunction 1 year following surgery, conferred worse long‐term prognosis.

### Prevalence and Interaction of Hepatorenal Dysfunction and Malnutrition in VHD

VHD not only adversely affects the myocardium but also is frequently associated with extracardiac pathophysiological consequences. Previous studies^10^, ^12^, ^13^, ^14^ have shown a substantial, albeit variable, prevalence of hepatorenal dysfunction (18%–45%) and malnutrition (42%–94%) in selected valvular procedures. Our study complements existing literature to demonstrate a high prevalence of hepatorenal dysfunction (24%) and malnutrition (61%) in a large and prospectively recruited valvular surgery cohort, and importantly highlights that these 2 conditions often occurred concomitantly (19%). Collectively, these results confirm the close interaction between the cardiovascular system and other organ systems and negate the notion that VHD is a single‐organ disease.

**Figure 4:**
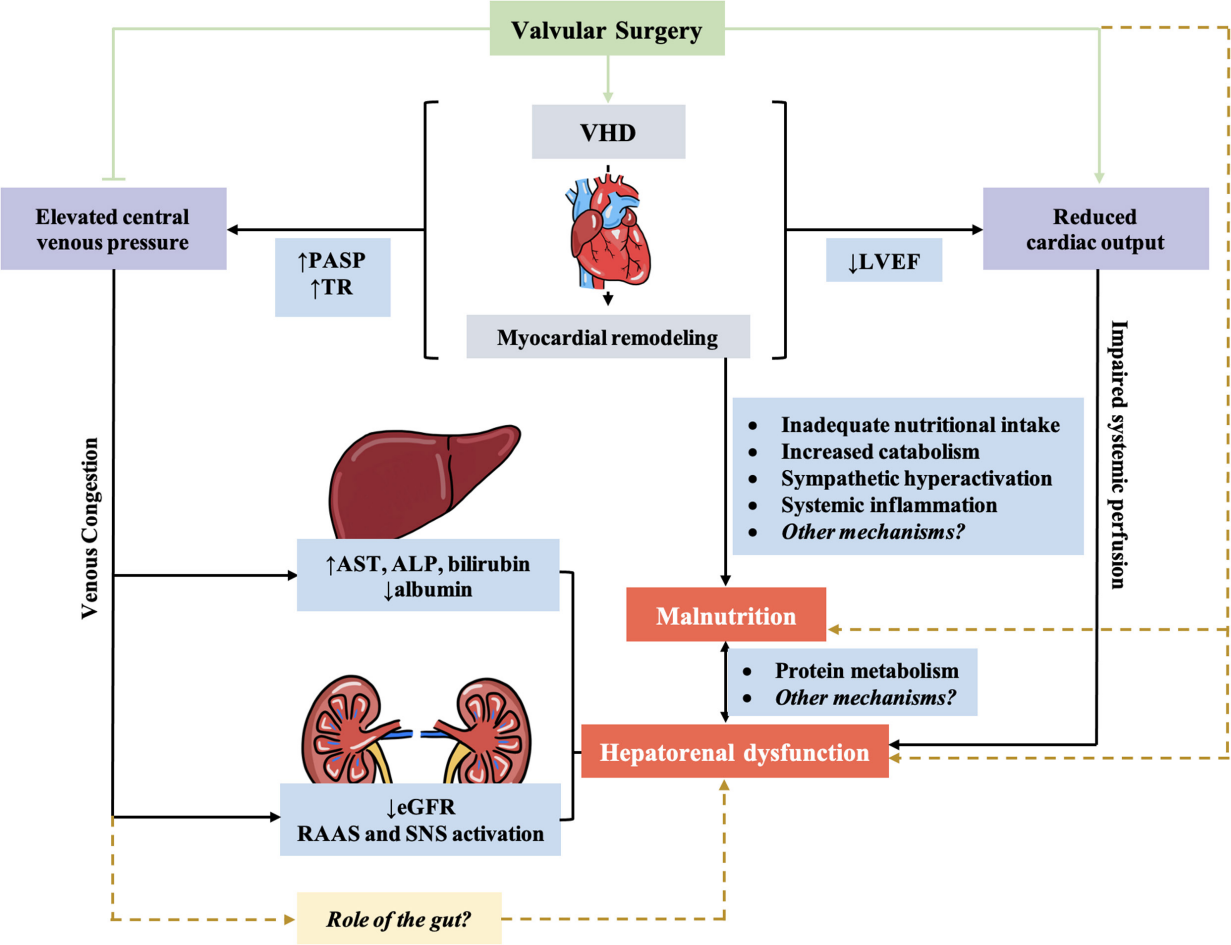
Mechanistic overview of the development of extracardiac sequelae (hepatorenal dysfunction and malnutrition) in valvular heart disease. ALP indicates alkaline phosphatase; AST, aspartate aminotransferase; eGFR, estimated glomerular filtration rate; LVEF, left ventricular ejection fraction; PASP, pulmonary artery systolic pressure; RAAS, renin‐angiotensin‐aldosterone system; SNS, sympathetic nervous system; and VHD, valvular heart disease.

There is a complex pathophysiological interaction between the heart and other organ systems. From a pathophysiological point of view, hepatorenal dysfunction develops from venous congestion associated with elevated right‐sided filling pressures,^20^ whereas malnutrition results from systemic inflammation common to LV dysfunction^21^ and pulmonary arterial hypertension,^22^ a notion supported by our observations of a lower left ventricular ejection fraction, higher pulmonary artery systolic pressure, and more prevalent TR in patients with hepatorenal dysfunction or malnutrition. Despite potential links between hepatorenal function and nutrition via protein metabolism,^23^ they are likely parallel manifestations that represent different downstream sequelae of VHD (Figure 4). The progressive increase in LV mass, pulmonary artery systolic pressure, and significant TR concordant with worsening hepatorenal function and nutrition further implies that their concomitant dysfunction represents an advanced stage of underlying VHD (more severe disease, longer duration, and late surgical referral) where forward and backward failures are key pathophysiologies in their development.

### Prognostic Role of Hepatorenal Function and Nutritional Status in Patients Undergoing Valvular Surgery

Although extracardiac organ systems may be considered a receptacle for valvular hemodynamics, evidence is mounting that end‐organ damage is not merely a passive bystander but also aggravates excess mortality and morbidity in VHD, even after successful intervention.^24^ In this context, hepatorenal dysfunction and malnutrition have emerged as meaningful prognosticators in valvular surgery, although current data^10^, ^12^, ^13^, ^14^ are limited to subpopulations with short follow‐up. The present study extends these observations for the first time to a large Asian VHD population and distinctly demonstrates a stepwise increase in the risk of 4 clinically relevant end points across a worsening spectrum of hepatorenal and nutritional phenotypes, independent of EuroSCORE II and STS Score. While putative mechanisms warrant clarification, their combined role in prognostication may be attributed to metabolic derangements, physical deconditioning, and poor wound healing characteristic of hepatorenal dysfunction and malnutrition,^8^, ^25^ with the latter being the most common cause of secondary immunologic dysfunction.^26^ Their concomitant dysfunction may also lead to an increased vulnerability to small and/or acute stresses^27^ that conceivably predispose patients to an escalated adverse outcome during major triggers such as valvular surgery. Accordingly, the combined assessment of hepatorenal function and nutritional status may identify individuals at high risk of HF and death and provide integral prognostic value beyond those captured by EuroSCORE II and STS score alone.

Furthermore, because of their interactions with the cardiovascular system, hepatorenal function and nutritional status do not remain static, and their postoperative assessment may thus inform the clinical course beyond that of baseline evaluation alone. Egbe et al^11^ showed an association between temporal deterioration of hepatorenal function and transplant‐free survival in patients with Ebstein anomaly, while Gonzalez Ferreiro et al^28^ found that patients remaining at nutritional risk after transcatheter aortic valve implantation exhibited an increased risk of mortality and HF hospitalization. Extending these findings, our study demonstrates that concomitant hepatorenal dysfunction and malnutrition persisted in a substantial proportion (15%) of patients undergoing valvular surgery. They had a poorer prognosis and thus they must be closely monitored during postoperative surveillance.

### Clinical Implications

Conventional risk‐scoring systems, particularly EuroSCORE II and STS score, have been widely used for long‐term risk stratification of patients undergoing valvular surgery.^3^, ^4^ Although these risk‐scoring systems provide good discriminatory power for perioperative mortality, they may serve only as initial estimates of long‐term prognosis that is nonetheless central to the lifelong management of patients with VHD. In line with previous studies,^5^, ^29^ our results showed that EuroSCORE II (area under the curve: 0.73) and STS score (area under the curve: 0.72) demonstrated only modest accuracy in predicting long‐term outcomes, an issue magnified by their tendency to underestimate mortality in high‐risk populations that will likely encompass increasingly comorbid patients with multiple valvular lesions.^5^, ^6^ In an attempt to optimize risk prediction in VHD, adjunctive measures such as exercise testing^30^ and biomarker profiling^31^ have been evaluated. These parameters, although prognostically meaningful, are costly and technical, and pose tremendous challenges to their clinical application. The incremental and discriminative prognostic value of MELD‐XI and CONUT scores, comprising simple and objective parameters obtained during routine assessment, thus offers an attractive alternative to aid conventional risk‐scoring systems. Moreover, their prognostic capacity even in specific valvular procedures and VHD origins provides compelling and generalizable evidence to support their use in the routine clinical setting. Beyond preoperative evaluation, MELD‐XI and CONUT scores at longitudinal follow‐up offer additional prognostic information that supports continual monitoring of these phenotypes postoperatively. As such, our study provides robust evidence to support an extracardiac workup in addition to EuroSCORE II and STS score for both pre‐ and postoperative clinical assessment. Accordingly, adopting a multiparametric approach can identify high‐risk surgical candidates with a dual burden of cardiac and extracardiac manifestations, who may instead opt for transcatheter valvular interventions as a low‐risk alternative. Still, the applicability of MELD and CONUT scores in transcatheter valvular therapies requires further study.

### Study Limitations

This was a single‐center, retrospective study and was thus subject to limitations inherent to this type of study design. The association between hepatorenal function, malnutrition, and adverse events could have been influenced by potential confounders, although extensive adjustments were made in multiple regression models. Our study evaluated hepatorenal function and nutritional status reflected by MELD‐XI and CONUT scores only and did not compare their prognostic value with either liver or kidney imaging data or more complex comprehensive nutritional assessments. Nonetheless, MELD‐XI and CONUT scores have been well validated^32^, ^33^ and showed strong correlation with clinical outcomes.^11^, ^12^, ^13^, ^14^, ^19^ Patients in the current study were Asians, with a large proportion having chronic rheumatic heart disease. Confirmatory studies in other ethnic groups and degenerative valvular pathologies are warranted, although subgroup analysis demonstrating their sustained prognostic capacity in patients with non–chronic rheumatic heart disease seems to support their utility in various causes of VHD.

## Conclusions

In a large cohort of patients undergoing valvular surgery, we demonstrated that hepatorenal dysfunction and malnutrition, as either isolated or combined phenotypes, are frequent and are associated with cardiac remodeling. Concomitant hepatorenal dysfunction and malnutrition at baseline, and their temporal deterioration at follow‐up, have a powerful, independent, and incremental link to excess mortality and worsening HF. Importantly, hepatorenal and nutritional assessment improves the prognostic and discriminatory power of existing valvular surgery risk‐scoring systems. These results highlight the prognostic importance of extracardiac organ‐system involvement in VHD and have major implications for optimizing risk‐stratification in patients undergoing valvular surgery.

## Sources of funding

This study is supported by the Sanming Project of Medicine in Shenzhen, China (No. SZSM201911020) and HKU‐SZH Fund for Shenzhen Key Medical Discipline (No. SZXK2020081).

## Disclosures

None.

## Supporting information

Tables S1–S11
Figures S1–S4
