# Supplementary material for: Concomitant Hepatorenal Dysfunction and Malnutrition in Valvular Heart Surgery: Long‐Term Prognostic Implications for Death and Heart Failure
Source: J Am Heart Assoc. 2022 May 16;11(10):e024060. doi: 10.1161/JAHA.121.024060 (PMC9238555; doi:10.1161/JAHA.121.024060)
Supplement: Supplementary file 1 — Tables S1–S11 Figures S1–S4 [file JAH3-11-e024060-s001.pdf]

SUPPLEMENTAL MATERIAL

Table S1. Baseline characteristics of patients included and excluded from the study

Characteristics	Patients included in the study (n=909)	Patients excluded from the study (n=171)	P value
**Demographic and anthropometric characteristics**			
Age, years	63 (57-69)	64 (50-72)	0.647
Male	431 (47.4)	91 (53.2)	0.320
Height, cm	159 (153-166)	164 (157-170)	0.002
Weight, kg	58 (50-67)	61 (52-67)	0.717
Body mass index, kg/m^2	23.0 (20.6-25.6)	22.6 (20.3-24.7)	0.103
NYHA Class III/IV	69 (7.6)	10 (5.8)	0.139
**Cardiovascular risk factors and cardiovascular disease**			
Hypertension	290 (31.9)	53 (31.0)	0.973
Diabetes Mellitus	165 (18.2)	24 (14.0)	0.194
Dyslipidemia	231 (25.4)	30 (17.5)	0.013
Smoking	178 (19.6)	37 (21.6)	0.808
Prior myocardial infarction	37 (4.1)	5 (2.9)	0.404
Prior stroke	82 (9.1)	15 (8.8)	0.867
Heart failure	393 (43.2)	58 (33.9)	0.032
Atrial fibrillation	487 (53.6)	71 (41.5)	0.011
**Comorbidities**			

Chronic obstructive pulmonary disease	52 (5.7)	7 (4.1)	0.479
Cancer	52 (5.7)	16 (9.4)	0.020
**Laboratory examination**			
Hemoglobin, g/dL	12.9 (11.6-14.0)	12.9 (11.3-14.1)	0.955
White cell count, x 10^9/L	5.9 (4.9-7.0)	8.3 (5.7-10.6)	<0.001
Platelet count, x 10^9/L	189 (157-227)	284 (191-356)	<0.001
Creatinine, mg/dL	0.92 (0.78-1.14)	–	–
eGFR, ml/min/1.73m^2	75.9 (60.9-90.1)	–	–
AST, U/L	27 (22-35)	–	–
ALT, U/L	21 (16-29)	–	–
ALP, U/L	71 (58-90)	–	–
Total bilirubin, mg/dL	0.53 (0.75-1.14)	–	–
Total cholesterol, mg/dL	159 (134-188)	–	–
Albumin, g/dL	4.2 (4.0-4.4)	–	–
**Valvular heart disease and echocardiographic variables**			
MS ≥ moderate	229 (25.2)	14 (8.2)	<0.001
MR ≥ moderate	411 (45.2)	112 (62.6)	0.071
AS ≥ moderate	322 (35.4)	33 (19.3)	<0.001
AR ≥ moderate	231 (25.4)	39 (39.2)	0.054
TR ≥ moderate	365 (40.2)	94 (54.9)	0.219

Chronic Rheumatic Heart Disease	259 (28.5)	35 (20.5)	0.052
LVEF, %	60 (55-60)	60 (55-60)	0.548
PASP, mmHg	40 (35-50)	45 (30-75)	0.019
**Medications**			
ACEI	286 (31.5)	37 (21.6)	0.009
ARB	142 (15.6)	24 (14.0)	0.679
Aldactone	117 (12.9)	23 (13.5)	0.977
Beta blockers	374 (41.1)	63 (36.8)	0.499
Calcium channel blockers	185 (20.4)	27 (15.8)	0.161
Digoxin	269 (29.6)	37 (21.6)	0.155
Statin	373 (41.0)	48 (28.1)	<0.001
Warfarin	417 (45.9)	58 (33.9)	0.005
**Cardiac surgery risk-stratification models**			
EuroScore II	2.42 (1.33-4.50)	1.68 (1.04-2.78)	<0.001
STS Score	1.49 (0.87-2.76)	0.91 (0.43-1.81)	<0.001
**Valvular surgery details**			
Aortic valve replacement	460 (50.7)	66 (38.6)	0.209
Mitral valve procedure	554 (61.0)	120 (70.2)	0.398
Mitral valve replacement	295 (32.5)	45 (26.3)	0.584
Mitral valve repair	259 (28.6)	75 (43.9)	0.021

Tricuspid annuloplasty	319 (35.2)	44 (25.7)	0.108
Concomitant CABG	107 (11.8)	14 (8.2)	0.287

Values are expressed as median (interquartile range) or number (percentage). P value by Mann-Whitney U test for non-normally distributed continuous variables; P value by χ^2 test for categorical variables.

Abbreviations: ACEI, angiotensin-converting enzyme inhibitors; ARB, angiotensin II receptor blockers; ALP, alkaline phosphatase; ALT, alanine aminotransaminase; AR, aortic regurgitation; AS, aortic stenosis; AST, aspartate aminotransferase; CABG, coronary artery bypass grafting; eGFR, estimated glomerular filtration rate; EuroSCORE II, European System for Cardiac Operative Risk Evaluation II; LVEF; left ventricular ejection fraction; PASP, pulmonary artery systolic pressure; MR, mitral regurgitation; MS, mitral stenosis; NYHA, New York Heart Association; STS score, Society of Thoracic Surgeons Predicted Risk of Mortality Score; TR, tricuspid regurgitation.

Table S2A. Univariate and multivariate logistic regression models showing predictors of baseline concomitant hepatorenal dysfunction (MELD-XI) and malnutrition (CONUT) (severe) in patients undergoing valvular surgery

	**Univariate analysis**		**Multivariate analysis (EuroSCORE II model)**		**Multivariate analysis (STS Score model)**	
	OR (95% CI)	P value	OR (95% CI)	P value	OR (95% CI)	P value
**Demographic and anthropometric characteristics**						
Age, years	1.04 (1.02-1.06)	<0.001				
Male	1.92 (1.38-2.70)	<0.001				
Body mass index, kg/m^2	0.96 (0.92-1.01)	0.087				
NYHA functional class III/IV	2.96 (1.75-4.92)	<0.001				
**Cardiovascular risk factors and cardiovascular disease**						
Hypertension	1.35 (0.95-1.90)	0.087				
Diabetes Mellitus	2.55 (1.74-3.71)	<0.001				
Smoking	1.36 (0.91-2.01)	0.122				
Dyslipidemia	1.08 (0.74-1.55)	0.698				
Prior myocardial infarction	1.56 (0.71-3.19)	0.240				
Prior stroke	1.84 (1.09-3.02)	0.019	1.33 (0.69-2.47)	0.378	1.67 (0.86-3.21)	0.131
Atrial fibrillation	2.23 (1.58-3.18)	<0.001	1.10 (0.67-1.83)	0.714	1.15 (0.70-1.89)	0.585
Heart failure	2.45 (1.76-3.45)	<0.001	1.43 (0.93-2.21)	0.108	1.42 (0.91-2.23)	0.123

**Valvular heart disease and echocardiographic variables**						
MS ≥ moderate	0.83 (0.55-1.24)	0.379				
MR ≥ moderate	1.40 (1.01-1.96)	0.045	1.15 (0.73-1.81)	0.548		
AS ≥ moderate	0.78 (0.54-1.12)	0.188				
AR ≥ moderate	0.86 (0.58-1.26)	0.450				
TR ≥ moderate	3.12 (2.22-4.40)	<0.001	1.93 (1.17-3.21)	0.010		
LV Mass, g	1.004 (1.002-1.005)	<0.001	1.005 (1.003-1.008)	<0.001	1.008 (1.005-1.010)	<0.001
LVEF, %	0.97 (0.95-0.98)	<0.001				
PASP, mmHg	1.03 (1.02-1.05)	<0.001	1.02 (1.01-1.04)	0.005	1.03 (1.01-1.04)	<0.001
**Cardiac surgery risk-stratification models**						
EuroSCORE II	1.16 (1.11-1.21)	<0.001	1.17 (1.11-1.24)	<0.001		
STS Score	1.45 (1.33-1.57)	<0.001			1.39 (1.26-1.53)	<0.001

Abbreviations: AR, aortic regurgitation; AS, aortic stenosis; CI, confidence interval; CONUT, Controlling Nutritional Status score; EuroSCORE II, European System for Cardiac Operative Risk Evaluation II; LV, left ventricle; LVEF, left ventricular ejection fraction; PASP, pulmonary artery systolic pressure; MELD-XI, Model for End-Stage Liver Disease excluding international normalized ratio; MR, mitral regurgitation; MS, mitral stenosis; NYHA, New York Heart Association; OR, odds ratio; STS score, Society of Thoracic Surgeons Predicted Risk of Mortality Score; TR, tricuspid regurgitation.

Table S2B. Competing Risk Analysis of baseline hepatorenal function (MELD-XI) and nutritional status (CONUT) for predicting heart failure hospitalization

	**Unadjusted**	**EuroSCORE II model***	**STS Score model†**
	SHR (95% CI)	SHR (95% CI)	SHR (95% CI)
**Combined evaluation of hepatorenal function and nutritional status**			
Normal (Normal hepatorenal function and well-nourished)	1.00	1.00	1.00
Mild (Hepatorenal dysfunction or malnutrition)	3.08 (1.78-5.35)	2.08 (1.23-3.52)	1.87 (1.08-3.24)
Severe (Hepatorenal dysfunction and malnutrition)	5.77 (3.26-10.23)	3.09 (1.73-5.50)	2.95 (1.59-5.47)

*Adjusted for atrial fibrillation, heart failure, significant tricuspid regurgitation, pulmonary artery systolic pressure, and EuroSCORE II (P<0.05 on univariate analysis).

†Adjusted for atrial fibrillation, heart failure, pulmonary artery systolic pressure, and STS Score (P<0.05 on univariate analysis).

Abbreviations: CI, confidence interval; CONUT, Controlling Nutritional Status score; EuroSCORE II, European System for Cardiac Operative Risk Evaluation II; MELD-XI, Model for End-Stage Liver Disease excluding international normalized ratio; SHR, subdistribution hazard ratio; STS score, Society of Thoracic Surgeons Predicted Risk of Mortality Score.

Table S2C. Competing Risk Analysis of baseline hepatorenal function (MELD-XI) and nutritional status (CONUT) for predicting cardiovascular death

	**Unadjusted**	**EuroSCORE II model***	**STS Score model†**
	SHR (95% CI)	SHR (95% CI)	SHR (95% CI)
**Combined evaluation of hepatorenal function and nutritional status**			
Normal (Normal hepatorenal function and well-nourished)	1.00	1.00	1.00
Mild (Hepatorenal dysfunction or malnutrition)	3.97 (1.36-11.6)	3.29 (1.14-9.52)	3.66 (1.24-10.82)
Severe (Hepatorenal dysfunction and malnutrition)	14.70 (5.17-41.7)	9.29 (3.09-27.99)	10.52 (3.49-31.74)

*Adjusted for hypertension, significant tricuspid regurgitation, and EuroSCORE II (P<0.05 on univariate analysis).

†Adjusted for hypertension and STS Score (P<0.05 on univariate analysis).

Abbreviations: CI, confidence interval; CONUT, Controlling Nutritional Status score; EuroSCORE II, European System for Cardiac Operative Risk Evaluation II; MELD-XI, Model for End-Stage Liver Disease excluding international normalized ratio; SHR, subdistribution hazard ratio; STS score, Society of Thoracic Surgeons Predicted Risk of Mortality Score.

Table S2D. Cox proportional hazards analyses of baseline hepatorenal function (MELD-XI) and nutritional status (CONUT) for predicting adverse events

	**Overall Population**					
	**Univariate analysis**		**Multivariate analysis (EuroSCORE II Model)**		**Multivariate analysis (STS Score Model)**	
	HR (95% CI)	P value	HR (95% CI)	P value	HR (95% CI)	P value
**Demographic and anthropometric characteristics**						
Age, years	1.05 (1.04-1.07)	<0.001				
Male	1.06 (0.79-1.40)	0.713				
Body mass index, kg/m^2	1.02 (0.98-1.06)	0.428				
NYHA Class III/IV	1.69 (1.09-2.64)	0.020				
**Cardiovascular risk factors and cardiovascular disease**						
Hypertension	1.52 (1.14-2.04)	0.005	1.61 (1.10-2.36)	0.013		
Diabetes Mellitus	2.41 (1.78-3.27)	<0.001				
Smoking	1.16 (0.82-1.64)	0.391				
Dyslipidemia	1.49 (1.10-2.02)	0.009	1.02 (0.69-1.51)	0.926	1.05 (0.72-1.54)	0.802
Prior myocardial infarction	2.16 (1.27-3.65)	0.004				
Prior stroke	1.91 (1.28-2.86)	0.002	1.33 (0.84-2.11)	0.217	1.39 (0.86-2.26)	0.177
Atrial fibrillation	1.63 (1.22-2.20)	0.001	1.01 (0.63-1.62)	0.957	1.19 (0.72-1.95)	0.497

Heart failure	2.45 (1.82-3.29)	<0.001	2.23 (1.55-3.20)	<0.001		
**Laboratory examination**						
Hemoglobin, g/dL	0.73 (0.68-0.79)	<0.001				
eGFR, ml/min/1.73m^2	0.974 (0.969-0.980)	<0.001				
Total bilirubin, mg/dL	1.32 (1.13-1.55)	<0.001				
Total cholesterol, mg/dL	0.987 (0.983-0.991)	<0.001				
Albumin, mg/dL	0.40 (0.29-0.56)	<0.001				
**Valvular heart disease and echocardiographic variables**						
MS ≥ moderate	1.12 (0.80-1.57)	0.519				
MR ≥ moderate	0.74 (0.55-0.99)	0.042	0.51 (0.36-0.73)	<0.001		
AS ≥ moderate	1.12 (0.83-1.53)	0.456				
AR ≥ moderate	0.80 (0.57-1.13)	0.199				
TR ≥ moderate	2.01 (1.51-2.67)	<0.001	1.44 (0.89-2.33)	0.138		
Chronic Rheumatic Heart Disease	1.39 (1.03-1.88)	0.031	1.15 (0.78-1.69)	0.474	0.96 (0.63-1.44)	0.829
LV Mass, g	1.001 (0.999-1.002)	0.328				
LVEF, %	0.97 (0.96-0.99)	<0.001				
PASP, mmHg	1.02 (1.01-1.02)	0.003	1.00 (0.99-1.01)	0.461	1.00 (0.99-1.01)	0.913
**Medications**						
ACEI	1.62 (1.21-2.16)	0.001	1.40 (0.99-1.97)	0.057	1.78 (1.26-2.51)	0.001

ARB	1.04 (0.70-1.53)	0.848				
Beta blockers	0.95 (0.71-1.28)	0.748				
Calcium channel blockers	0.97 (0.68-1.39)	0.884				
Digoxin	1.42 (1.06-1.91)	0.019				
Statin	1.27 (0.95-1.69)	0.101				
Warfarin	1.68 (1.26-2.23)	<0.001	1.11 (0.70-1.76)	0.645	0.96 (0.60-1.54)	0.866
**Valvular surgery details**						
Aortic valve replacement	1.13 (0.85-1.51)	0.395				
Mitral valve procedure	0.99 (0.74-1.33)	0.958				
Mitral valve replacement	1.10 (0.82-1.48)	0.547				
Mitral valve repair	0.91 (0.65-1.25)	0.527				
Tricuspid annuloplasty	1.66 (1.25-2.21)	<0.001	1.06 (0.67-1.67)	0.797	1.24 (0.84-1.82)	0.282
Concomitant CABG	1.27 (0.85-1.91)	0.248				
**Cardiac surgery risk-stratification models**						
EuroScore II	1.06 (1.05-1.07)	<0.001	1.04 (1.02-1.06)	<0.001		
STS Score	1.26 (1.20-1.32)	<0.001			1.16 (1.09-1.23)	<0.001

**Combined evaluation of hepatorenal function and nutritional status**						
Normal (Normal hepatorenal function and well-nourished)	1.00		1.00		1.00	
Mild (Hepatorenal dysfunction or malnutrition)	3.19 (2.03-4.99)	<0.001	2.11 (1.25-3.55)	0.005	1.97 (1.18-3.32)	0.010
Severe (Hepatorenal dysfunction and malnutrition)	7.22 (4.56-11.44)	<0.001	3.55 (2.04-6.16)	<0.001	3.17 (1.80-5.62)	<0.001

Abbreviations: ACEI, angiotensin-converting enzyme inhibitors; ARB, angiotensin II receptor blockers; ALP, alkaline phosphatase; ALT, alanine aminotransaminase; AR, aortic regurgitation; AS, aortic stenosis; AST, aspartate aminotransferase; CABG, coronary artery bypass grafting; CI, confidence interval; CONUT, Controlling Nutritional Status score; eGFR, estimated glomerular filtration rate; EuroSCORE II, European System for Cardiac Operative Risk Evaluation II; HR, hazard ratio; LVEF, left ventricular ejection fraction; PASP, pulmonary artery systolic pressure; MELD-XI, Model for End-Stage Liver Disease excluding international normalized ratio; MR, mitral regurgitation; MS, mitral stenosis; NYHA, New York Heart Association; STS score, Society of Thoracic Surgeons Predicted Risk of Mortality Score; TR, tricuspid regurgitation.

Table S3A. Cox proportional hazards analyses of baseline hepatorenal function (MELD-XI) and nutritional status (CONUT) for predicting all-cause mortality and adverse events in patients undergoing aortic valve replacement (n=460)

	**Multivariate analysis**							
	**EuroSCORE II model**				**STS Score model**			
	All-cause mortality*		Adverse events†		All-cause mortality‡		Adverse events§	
	HR (95% CI)	P value	HR (95% CI)	P value	HR (95% CI)	P value	HR (95% CI)	P value
**Separate evaluation of hepatorenal function and nutritional status**								
<u>Hepatorenal dysfunction</u>								
MELD-XI, continuous	1.14 (1.09-1.19)	<0.001	1.07 (1.03-1.12)	0.002	1.12 (1.07-1.18)	<0.001	1.08 (1.02-1.13)	0.004
MELD-XI, categorical								
Hepatorenal dysfunction (MELD-XI >12.43)	2.96 (1.69- 5.16)	<0.001	2.28 (1.42-3.67)	<0.001	3.16 (1.79-5.59)	<0.001	2.39 (1.43-4.02)	<0.001
<u>Nutritional status</u>								
CONUT, continuous	1.43 (1.24-1.66)	<0.001	1.32 (1.14-1.53)	<0.001	1.46 (1.27-1.68)	<0.001	1.31 (1.13-1.52)	<0.001
CONUT, categorical								
Normal nutrition	Referent		Referent		Referent		Referent	

Mild malnutrition	3.97 (1.76-8.93)	<0.001	2.88 (1.47-5.64)	0.002	4.72 (1.97-11.30)	<0.001	3.10 (1.52-6.34)	0.002
Moderate to severe malnutrition	8.07 (2.98-21.84)	<0.001	5.73 (2.40-13.64)	<0.001	10.37 (3.68-29.26)	<0.001	5.78 (2.34-14.28)	<0.001
**Combined evaluation of hepatorenal function and nutritional status**								
Normal (Normal hepatorenal function and well-nourished)	Referent		Referent		Referent		Referent	
Mild (Hepatorenal dysfunction or malnutrition)	4.36 (1.68-11.33)	0.003	2.56 (1.25-5.23)	0.010	4.40 (1.68-11.52)	0.003	2.49 (1.20-5.19)	0.014
Severe (Hepatorenal dysfunction and malnutrition)	9.50 (3.49-25.85)	<0.001	4.76 (2.24-10.13)	<0.001	10.57 (3.88-28.85)	<0.001	5.15 (2.35-11.27)	<0.001

*Adjusted for hypertension, heart failure, concomitant mitral valve procedure, concomitant tricuspid annuloplasty, and EuroSCORE II ($P<0.05$ on univariate analysis).

†Adjusted for hypertension, atrial fibrillation, prior stroke, baseline warfarin therapy, pulmonary artery systolic pressure, concomitant mitral valve procedure, concomitant tricuspid annuloplasty, and EuroSCORE II ($P<0.05$ on univariate analysis).

‡Adjusted for concomitant mitral valve procedure, concomitant tricuspid annuloplasty, and STS Score ($P<0.05$ on univariate analysis).

§Adjusted for atrial fibrillation, prior stroke, baseline warfarin therapy, pulmonary artery systolic pressure, concomitant mitral valve procedure, concomitant tricuspid annuloplasty, and EuroSCORE II ($P<0.05$ on univariate analysis).

Abbreviations: CI, confidence interval; CONUT, Controlling Nutritional Status score; EuroSCORE II, European System for Cardiac Operative Risk Evaluation II; HR, hazard ratio; MELD-XI, Model for End-Stage Liver Disease excluding international normalized ratio; STS score, Society of Thoracic Surgeons Predicted Risk of Mortality Score.

Table S3B. Cox proportional hazards analyses of baseline hepatorenal function (MELD-XI) and nutritional status (CONUT) for predicting all-cause mortality and adverse events in patients undergoing mitral valve procedure (mitral valve replacement or mitral valve repair) (n=554)

	**Multivariate analysis**							
	**EuroSCORE II model**				**STS Score model**			
	All-cause mortality*		Adverse events†		All-cause mortality‡		Adverse events§	
	HR (95% CI)	P value	HR (95% CI)	P value	HR (95% CI)	P value	HR (95% CI)	P value
**Separate evaluation of hepatorenal function and nutritional status**								
Hepatorenal dysfunction								
MELD-XI, continuous	1.12 (1.08-1.17)	<0.001	1.06 (1.02-1.10)	0.002	1.11 (1.06-1.16)	<0.001	1.04 (1.00-1.08)	0.034
MELD-XI, categorical								
Hepatorenal dysfunction (MELD-XI >12.43)	4.13 (2.23-7.63)	<0.001	1.85 (1.25-2.74)	0.002	3.85 (2.02-7.32)	<0.001	1.72 (1.14-2.59)	0.012
Nutritional status								
CONUT, continuous	1.61 (1.34-1.93)	<0.001	1.33 (1.18-1.50)	<0.001	1.54 (1.28-1.86)	<0.001	1.30 (1.15-1.47)	<0.001
CONUT, categorical								
Normal nutrition	Referent		Referent		Referent		Referent	

Mild malnutrition	7.16 (2.16-23.70)	0.001	2.13 (1.28-3.52)	0.004	6.75 (2.03-22.45)	0.002	1.94 (1.16-3.26)	0.012
Moderate to severe malnutrition	20.24 (5.55-73.82)	<0.001	5.33 (2.84-9.99)	<0.001	15.34 (4.08-57.60)	<0.001	4.39 (2.30-8.39)	<0.001
**Combined evaluation of hepatorenal function and nutritional status**								
Normal (Normal hepatorenal function and well-nourished)	Referent		Referent		Referent		Referent	
Mild (Hepatorenal dysfunction or malnutrition)	3.71 (1.06-12.93)	0.040	2.14 (1.22-3.75)	0.008	3.55 (1.01-12.46)	0.048	2.01 (1.14-3.55)	0.016
Severe (Hepatorenal dysfunction and malnutrition)	14.81 (4.37-50.24)	<0.001	3.60 (1.99-6.51)	<0.001	13.17 (3.83-45.29)	<0.001	3.16 (1.71-5.84)	<0.001

*Adjusted for hypertension, concomitant aortic valve replacement, concomitant tricuspid annuloplasty, and EuroSCORE II (P<0.05 on univariate analysis).

†Adjusted for hypertension, dyslipidemia, prior myocardial infarction, concomitant aortic valve replacement, concomitant tricuspid annuloplasty, concomitant coronary artery bypass grafting, and EuroSCORE II ($P<0.05$ on univariate analysis).

‡Adjusted for concomitant aortic valve replacement, concomitant tricuspid annuloplasty, and STS Score ($P<0.05$ on univariate analysis).

§Adjusted for dyslipidemia, prior myocardial infarction, concomitant aortic valve replacement, concomitant tricuspid annuloplasty, concomitant coronary artery bypass grafting, and STS Score ($P<0.05$ on univariate analysis).

Abbreviations: CI, confidence interval; CONUT, Controlling Nutritional Status score; EuroSCORE II, European System for Cardiac Operative Risk Evaluation II; HR, hazard ratio; MELD-XI, Model for End-Stage Liver Disease excluding international normalized ratio; STS score, Society of Thoracic Surgeons Predicted Risk of Mortality Score.

Table S3C. Cox proportional hazards analyses of baseline hepatorenal function (MELD-XI) and nutritional status (CONUT) for predicting all-cause mortality and adverse events in valvular heart disease of rheumatic etiology (n=259)

	**Multivariate analysis**							
	**EuroSCORE II model**				**STS Score model**			
	All-cause mortality*		Adverse events†		All-cause mortality‡		Adverse events§	
	HR (95% CI)	P value	HR (95% CI)	P value	HR (95% CI)	P value	HR (95% CI)	P value
**Separate evaluation of hepatorenal function and nutritional status**								
Hepatorenal dysfunction								
MELD-XI, continuous	1.16 (1.09-1.25)	<0.001	1.10 (1.03-1.17)	0.004	1.28 (1.12-1.47)	<0.001	1.12 (1.01-1.24)	0.032
MELD-XI, categorical								
Hepatorenal dysfunction (MELD-XI >12.43)	3.58 (1.82-7.06)	<0.001	1.89 (1.13-3.14)	0.015	3.72 (1.69-8.18)	0.001	1.86 (1.04-3.32)	0.035
Nutritional status								
CONUT, continuous	1.90 (1.50-2.41)	<0.001	1.44 (1.22-1.70)	<0.001	1.93 (1.45-2.56)	<0.001	1.34 (1.10-1.63)	0.003
CONUT, categorical								
Normal nutrition	Referent		Referent		Referent		Referent	

Mild malnutrition	9.70 (2.15-43.84)	0.003	4.87 (2.26-10.48)	<0.001	6.91 (1.60-29.84)	0.010	3.47 (1.60-7.52)	0.002
Moderate to severe malnutrition	32.94 (6.51-166.67)	<0.001	6.77 (2.60-17.64)	<0.001	22.90 (4.33-121.28)	<0.001	4.10 (1.33-12.65)	0.014
**Combined evaluation of hepatorenal function and nutritional status**								
Normal (Normal hepatorenal function and well-nourished)	Referent		Referent		Referent		Referent	
Mild (Hepatorenal dysfunction or malnutrition)	5.85 (1.30-26.38)	0.022	5.03 (2.16-11.69)	<0.001	4.60 (1.02-20.72)	0.047	3.36 (1.45-7.77)	0.005
Severe (Hepatorenal dysfunction and malnutrition)	15.22 (3.53-65.73)	<0.001	5.99 (2.55-14.07)	<0.001	14.64 (3.20-66.88)	<0.001	4.91 (1.98-12.20)	<0.001

*Adjusted for EuroSCORE II (P<0.05 on univariate analysis).

†Adjusted for heart failure, mitral valve replacement, and EuroSCORE II ($P<0.05$ on univariate analysis).

‡Adjusted for STS Score ($P<0.05$ on univariate analysis).

§Adjusted for mitral valve replacement and STS Score ($P<0.05$ on univariate analysis).

Abbreviations: CI, confidence interval; CONUT, Controlling Nutritional Status score; EuroSCORE II, European System for Cardiac Operative Risk Evaluation II; HR, hazard ratio; MELD-XI, Model for End-Stage Liver Disease excluding international normalized ratio; STS score, Society of Thoracic Surgeons Predicted Risk of Mortality Score.

Table S3D. Cox proportional hazards analyses of baseline hepatorenal function (MELD-XI) and nutritional status (CONUT) for predicting all-cause mortality and adverse events in valvular heart disease of non-rheumatic etiology (n=650)

	**Multivariate analysis**							
	**EuroSCORE II model**				**STS Score model**			
	All-cause mortality*		Adverse events†		All-cause mortality‡		Adverse events§	
	HR (95% CI)	P value	HR (95% CI)	P value	HR (95% CI)	P value	HR (95% CI)	P value
**Separate evaluation of hepatorenal function and nutritional status**								
Hepatorenal dysfunction								
MELD-XI, continuous	1.13 (1.09-1.17)	<0.001	1.09 (1.05-1.13)	<0.001	1.08 (1.03-1.13)	<0.001	1.04 (1.00-1.08)	0.057
MELD-XI, categorical								
Hepatorenal dysfunction (MELD-XI >12.43)	3.08 (1.81-5.24)	<0.001	2.03 (1.36-3.02)	<0.001	2.38 (1.33-4.24)	0.003	1.80 (1.17-2.78)	0.008
Nutritional status								
CONUT, continuous	1.44 (1.25-1.66)	<0.001	1.33 (1.19-1.48)	<0.001	1.39 (1.20-1.61)	<0.001	1.30 (1.17-1.45)	<0.001
CONUT, categorical								
Normal nutrition	Referent		Referent		Referent		Referent	

Mild malnutrition	2.99 (1.37-6.54)	0.006	1.89 (1.14-3.11)	0.013	3.03 (1.32-6.97)	0.009	1.71 (1.02-2.88)	0.043
Moderate to severe malnutrition	8.47 (3.43-20.93)	<0.001	4.80 (2.64-8.75)	<0.001	6.79 (2.61-17.67)	<0.001	3.95 (2.07-7.55)	<0.001
**Combined evaluation of hepatorenal function and nutritional status**								
Normal (Normal hepatorenal function and well-nourished)	Referent		Referent		Referent		Referent	
Mild (Hepatorenal dysfunction or malnutrition)	2.88 (1.17-7.10)	0.021	2.13 (1.21-3.76)	0.009	2.69 (1.09-6.67)	0.032	1.97 (1.11-3.50)	0.020
Severe (Hepatorenal dysfunction and malnutrition)	7.88 (3.12-19.95)	<0.001	3.81 (2.08-6.99)	<0.001	5.88 (2.28-15.17)	<0.001	3.13 (1.65-5.97)	<0.001

*Adjusted for hypertension, dyslipidemia, significant tricuspid regurgitation, aortic valve replacement, and EuroSCORE II (P<0.05 on univariate analysis).

†Adjusted for hypertension, dyslipidemia, atrial fibrillation, heart failure, baseline medical therapy (statin and warfarin), type of valvular surgery, and EuroSCORE II ($P<0.05$ on univariate analysis).

‡Adjusted for dyslipidemia, aortic valve replacement, and STS Score ($P<0.05$ on univariate analysis).

§Adjusted for dyslipidemia, atrial fibrillation, baseline medical therapy (statin and warfarin), type of valvular surgery, and STS Score ($P<0.05$ on univariate analysis).

Abbreviations: CI, confidence interval; CONUT, Controlling Nutritional Status score; EuroSCORE II, European System for Cardiac Operative Risk Evaluation II; HR, hazard ratio; MELD-XI, Model for End-Stage Liver Disease excluding international normalized ratio; STS score, Society of Thoracic Surgeons Predicted Risk of Mortality Score.

Table S4. Cox proportional hazards analyses of baseline hepatorenal function (MELD-XI) and nutritional status (CONUT) for predicting all-cause mortality and adverse events at least 30 days after valvular surgery (n=892)

	**Multivariate analysis**							
	**EuroSCORE II model**				**STS Score model**			
	All-cause mortality*		Adverse events†		All-cause mortality‡		Adverse events§	
	HR (95% CI)	P value	HR (95% CI)	P value	HR (95% CI)	P value	HR (95% CI)	P value
**Separate evaluation of hepatorenal function and nutritional status**								
Hepatorenal dysfunction								
MELD-XI, continuous	1.15 (1.11-1.19)	<0.001	1.08 (1.04-1.12)	<0.001	1.12 (1.08-1.17)	<0.001	1.06 (1.02-1.10)	0.006
MELD-XI, categorical								
Hepatorenal dysfunction (MELD-XI >12.43)	3.03 (1.90-4.81)	<0.001	1.69 (1.17-2.42)	0.005	2.81 (1.69-4.68)	<0.001	1.51 (1.02-2.24)	0.041
Nutritional status								
CONUT, continuous	1.41 (1.24-1.61)	<0.001	1.29 (1.16-1.42)	<0.001	1.44 (1.27-1.64)	<0.001	1.25 (1.12-1.39)	<0.001
CONUT, categorical								
Normal nutrition	Referent		Referent		Referent		Referent	

Mild malnutrition	3.79 (1.78-8.08)	<0.001	2.05 (1.28-3.26)	0.003	4.47 (2.00-10.00)	<0.001	1.96 (1.21-3.20)	0.007
Moderate to severe malnutrition	8.88 (3.74-21.06)	<0.001	4.51 (2.57-7.91)	<0.001	10.58 (4.19-26.70)	<0.001	3.79 (2.05-7.00)	<0.001
**Combined evaluation of hepatorenal function and nutritional status**								
Normal (Normal hepatorenal function and well-nourished)	Referent		Referent		Referent		Referent	
Mild (Hepatorenal dysfunction or malnutrition)	2.88 (1.26-6.59)	0.012	2.15 (1.28-3.62)	0.004	3.03 (1.32-6.97)	0.009	1.97 (1.16-3.35)	0.013
Severe (Hepatorenal dysfunction and malnutrition)	8.19 (3.53-18.99)	<0.001	3.26 (1.87-5.68)	<0.001	8.48 (3.60-20.00)	<0.001	2.83 (1.57-5.10)	<0.001

*Adjusted for hypertension, dyslipidemia, prior stroke, significant tricuspid regurgitation, type of valvular surgery, and EuroSCORE II (P<0.05 on univariate analysis).

†Adjusted for hypertension, dyslipidemia, atrial fibrillation, heart failure, prior myocardial infarction, prior stroke, baseline medical therapy (angiotensin-converting enzyme inhibitors, warfarin), significant tricuspid regurgitation, pulmonary artery systolic pressure, type of valvular surgery, and EuroSCORE II ($P<0.05$ on univariate analysis).

‡ Adjusted for dyslipidemia, prior stroke, type of valvular surgery, and STS Score ($P<0.05$ on univariate analysis).

§Adjusted for dyslipidemia, prior myocardial infarction, prior stroke, atrial fibrillation, baseline medical therapy (angiotensin-converting enzyme inhibitors, warfarin), pulmonary artery systolic pressure, type of valvular surgery, and STS Score ($P<0.05$ on univariate analysis).

Abbreviations: CI, confidence interval; CONUT, Controlling Nutritional Status score; EuroSCORE II, European System for Cardiac Operative Risk Evaluation II; HR, hazard ratio; MELD-XI, Model for End-Stage Liver Disease excluding international normalized ratio; STS score, Society of Thoracic Surgeons Predicted Risk of Mortality Score.

Table S5A. Baseline characteristics of the study population according to hepatorenal function (MELD-XI)

Characteristics	Overall (n=909)	Normal hepatorenal function (MELD-XI ≤12.43) (n=693)	Hepatorenal dysfunction (MELD-XI >12.43) (n=216)	P value
**Demographic and anthropometric characteristics**				
Age, years	63 (57-69)	62 (56-68)	64 (59-70)	<0.001
Male	431 (47.4)	295 (42.6)	136 (63.0)	<0.001
Height, cm	159 (153-166)	159 (153-165)	160 (154-167)	0.158
Weight, kg	58 (50-67)	58 (51-67)	58 (50-68)	0.857
Body mass index, kg/m^2	23.0 (20.6-25.6)	23.2 (20.7-25.7)	22.7 (19.9-25.4)	0.149
NYHA Class III/IV	69 (7.6)	40 (5.8)	29 (13.4)	<0.001
**Cardiovascular risk factors and cardiovascular disease**				
Hypertension	290 (31.9)	209 (30.2)	81 (37.5)	0.045
Diabetes Mellitus	165 (18.2)	104 (15.0)	64 (28.2)	<0.001
Dyslipidemia	231 (25.4)	171 (24.7)	60 (27.8)	0.371
Smoking	178 (19.6)	123 (17.7)	55 (25.5)	0.014
Prior myocardial infarction	37 (4.1)	24 (3.5)	13 (6.0)	0.114
Prior stroke	82 (9.1)	54 (7.8)	28 (13.1)	0.022
Heart failure	393 (43.2)	260 (37.5)	133 (61.6)	<0.001
Atrial fibrillation	487 (53.6)	343 (49.5)	144 (66.7)	<0.001
**Comorbidities**				

Chronic obstructive pulmonary disease	52 (5.7)	39 (5.6)	13 (6.0)	0.867
Cancer	52 (5.7)	42 (6.1)	10 (4.6)	0.504
**Laboratory examination**				
Hemoglobin, g/dL	12.9 (11.6-14)	13.1 (12-14)	11.8 (10.2-13.5)	<0.001
White cell count, x 10^9/L	5.9 (4.9-7.0)	5.9 (4.9-6.8)	6.0 (4.8-7.6)	0.292
Platelet count, x 10^9/L	189 (157-227)	192 (165-228)	177 (134-222)	<0.001
Creatinine, mg/dL	0.92 (0.78-1.14)	0.86 (0.75-1.01)	1.37 (1.12-1.54)	<0.001
eGFR, ml/min/1.73m^2	75.9 (60.9-90.1)	81.5 (69.8-93.5)	51.8 (38.0-62.4)	<0.001
AST, U/L	27 (22-35)	26 (22-33)	30 (25-40)	<0.001
ALT, U/L	21 (16-29)	21 (16-29)	20 (16-27)	0.167
ALP, U/L	71 (58-90)	70 (56-85)	82 (63-113)	<0.001
Total bilirubin, mg/dL	0.75 (0.53-1.14)	0.67 (0.51-0.94)	1.5 (0.76-2.13)	<0.001
Total cholesterol, mg/dL	159 (134-188)	164 (139-193)	142 (120-165)	<0.001
Albumin, g/dL	4.2 (4-4.4)	4.2 (4-4.4)	4 (3.7-4.3)	<0.001
**Valvular heart disease and echocardiographic variables**				
MS ≥ moderate	229 (25.2)	182 (30.7)	47 (25.7)	0.196
MR ≥ moderate	411 (45.2)	294 (43.0)	17 (54.7)	0.003
AS ≥ moderate	322 (35.4)	254 (40.5)	68 (34.9)	0.179
AR ≥ moderate	231 (25.4)	183 (27.6)	48 (22.6)	0.179
TR ≥ moderate	365 (40.2)	242 (34.9)	123 (57.2)	<0.001

Chronic Rheumatic Heart Disease	259 (28.5)	200 (28.9)	59 (27.3)	0.724
LV Mass, g	224 (176-294)	218 (170-283)	248 (194-314)	<0.001
LVEF, %	60 (55-60)	60 (55-60)	55 (50-60)	<0.001
Preserved, ≥50%	763 (84.3)	599 (86.6)	164 (77.0)	0.001
Mid-range, 40% - 49%	57 (6.3)	45 (6.5)	12 (5.6)	0.748
Reduced, <40%	55 (6.1)	29 (4.2)	26 (12.2)	<0.001
PASP, mmHg	40 (35-50)	40 (35-50)	45 (40-55)	<0.001
**Medications**				
ACEI	286 (31.5)	204 (29.4)	82 (38.0)	0.019
ARB	142 (15.6)	108 (15.6)	34 (15.7)	1.000
Aldactone	117 (12.9)	67 (9.7)	50 (23.1)	<0.001
Beta blockers	374 (41.1)	264 (38.1)	110 (50.9)	0.001
Calcium channel blockers	185 (20.4)	143 (20.6)	42 (19.4)	0.772
Digoxin	269 (29.6)	183 (26.4)	86 (39.8)	<0.001
Statin	373 (41)	274 (39.5)	99 (45.8)	0.113
Warfarin	417 (45.9)	300 (43.3)	117 (54.2)	0.006
**Cardiac surgery risk-stratification models**				
EuroScore II	2.4 (1.3-4.5)	2.1 (1.2-3.8)	4.2 (2.5-7.8)	<0.001
STS Score	1.5 (0.9-2.8)	1.4 (0.8-2.3)	2.5 (1.2-4.8)	<0.001
**Valvular surgery details**				

Aortic valve replacement	460 (50.7)	363 (52.4)	97 (45.1)	0.072
Mitral valve procedure	554 (61)	410 (59.2)	144 (66.7)	0.055
Mitral valve replacement	295 (32.5)	226 (32.6)	69 (31.9)	0.868
Mitral valve repair	259 (28.6)	184 (26.6)	75 (35.0)	0.019
Tricuspid annuloplasty	319 (35.2)	215 (31.0)	104 (48.6)	<0.001
Concomitant CABG	107 (11.8)	71 (10.3)	36 (16.8)	0.011

Values are expressed as median (interquartile range) or number (percentage). P value by Mann-Whitney U test for non-normally distributed continuous variables; P value by χ^2 test for categorical variables.

Abbreviations: ACEI, angiotensin-converting enzyme inhibitors; ARB, angiotensin II receptor blockers; ALP, alkaline phosphatase; ALT, alanine aminotransaminase; AR, aortic regurgitation; AS, aortic stenosis; AST, aspartate aminotransferase; CABG, coronary artery bypass grafting; eGFR, estimated glomerular filtration rate; EuroSCORE II, European System for Cardiac Operative Risk Evaluation II; LVEF; left ventricular ejection fraction; PASP, pulmonary artery systolic pressure; MR, mitral regurgitation; MS, mitral stenosis; NYHA, New York Heart Association; STS score, Society of Thoracic Surgeons Predicted Risk of Mortality Score; TR, tricuspid regurgitation.

Table S5B. Univariate and multivariate logistic regression models showing predictors of baseline hepatorenal dysfunction (MELD-XI) in patients undergoing valvular surgery

	**Univariate analysis**		**Multivariate analysis (EuroSCORE II model)**		**Multivariate analysis (STS Score model)**	
	OR (95% CI)	P value	OR (95% CI)	P value	OR (95% CI)	P value
**Demographic and anthropometric characteristics**						
Age, years	1.03 (1.01-1.05)	<0.001				
Male	2.29 (1.68-3.15)	<0.001				
Body mass index, kg/m^2	0.97 (0.93-1.01)	0.166				
NYHA functional class III/IV	2.53 (1.52-4.18)	<0.001				
**Cardiovascular risk factors and cardiovascular disease**						
Hypertension	1.39 (1.01-1.91)	0.044	1.10 (0.69-1.75)	0.674		
Diabetes Mellitus	2.23 (1.55-3.20)	<0.001				
Smoking	1.58 (1.10-2.27)	0.013	2.12 (1.26-3.53)	0.004	2.56 (1.50-4.38)	<0.001
Dyslipidemia	1.17 (0.83-1.65)	0.361				
Prior myocardial infarction	1.79 (0.87-3.52)	0.101	0.98 (0.33-2.75)	0.973	0.80 (0.27-2.28)	0.689
Prior stroke	1.77 (1.08-2.85)	0.021	1.40 (0.74-2.61)	0.297	1.33 (0.66-2.62)	0.422
Atrial fibrillation	2.04 (1.49-2.82)	<0.001	1.27 (0.78-2.09)	0.342	1.31 (0.81-2.15)	0.268
Heart failure	2.67 (1.95-3.66)	<0.001	1.63 (1.06-2.52)	0.026		

**Valvular heart disease and echocardiographic variables**						
MS ≥ moderate	0.78 (0.53-1.13)	0.195				
MR ≥ moderate	1.60 (1.18-2.18)	0.003	1.97 (1.25-3.13)	0.004		
AS ≥ moderate	0.78 (0.56-1.10)	0.159				
AR ≥ moderate	0.77 (0.53-1.10)	0.155				
TR ≥ moderate	2.49 (1.83-3.41)	<0.001	1.19 (0.72-1.95)	0.503		
LV Mass, g	1.004 (1.002-1.005)	<0.001	1.004 (1.001-1.006)	0.002	1.007 (1.004-1.009)	<0.001
LVEF, %	0.96 (0.95-0.98)	<0.001				
PASP, mmHg	1.03 (1.02-1.04)	<0.001	1.01 (1.00-1.03)	0.071	1.02 (1.01-1.03)	0.007
**Cardiac surgery risk-stratification models**						
EuroSCORE II	1.14 (1.10-1.19)	<0.001	1.11 (1.06-1.18)	<0.001		
STS Score	1.39 (1.28-1.50)	<0.001			1.26 (1.15-1.40)	<0.001
**Nutritional status**						
CONUT, continuous	1.76 (1.58-1.97)	<0.001	1.62 (1.41-1.88)	<0.001	1.64 (1.42-1.91)	<0.001

Abbreviations: AR, aortic regurgitation; AS, aortic stenosis; CI, confidence interval; CONUT, Controlling Nutritional Status score; EuroSCORE II, European System for Cardiac Operative Risk Evaluation II; LV, left ventricle; LVEF, left ventricular ejection fraction; PASP, pulmonary artery systolic pressure; MELD-XI, Model for End-Stage Liver Disease excluding international normalized ratio; MR, mitral regurgitation; MS, mitral stenosis; NYHA, New York Heart Association; OR, odds ratio; STS score, Society of Thoracic Surgeons Predicted Risk of Mortality Score; TR, tricuspid regurgitation.

Table S5C. Cox proportional hazards analysis of baseline hepatorenal function (MELD-XI) for predicting all-cause mortality and adverse events

	Multivariate analysis							
	EuroSCORE II model				STS Score model			
	All-cause mortality*		Adverse events†		All-cause mortality‡		Adverse events§	
	HR (95% CI)	P value	HR (95% CI)	P value	HR (95% CI)	P value	HR (95% CI)	P value
**Hepatorenal function**								
MELD-XI, continuous	1.14 (1.10-1.17)	<0.001	1.09 (1.05-1.12)	<0.001	1.11 (1.07-1.16)	<0.001	1.05 (1.01-1.09)	0.010
MELD-XI, categorical								
Normal hepatorenal function	1.00		1.00		1.00		1.00	
Hepatorenal dysfunction (MELD-XI >12.43)	3.35 (2.20-5.12)	<0.001	1.88 (1.32-2.67)	<0.001	2.94 (1.84-4.70)	<0.001	1.70 (1.17-2.48)	0.006

*Adjusted for hypertension, dyslipidemia, prior stroke, baseline warfarin therapy, type of valvular lesion, type of valvular surgery, EuroSCORE II (P<0.05 on univariate analysis).

†Adjusted for hypertension, dyslipidemia, prior stroke, atrial fibrillation, heart failure, baseline medical therapy (angiotensin-converting enzyme inhibitors, warfarin), chronic rheumatic heart disease, type of valvular lesion, tricuspid annuloplasty, pulmonary artery systolic pressure and EuroSCORE II (P<0.05 on univariate analysis).

‡Adjusted for dyslipidemia, prior stroke, baseline warfarin therapy, type of valvular surgery, and STS Score ($P<0.05$ on univariate analysis).

§Adjusted for dyslipidemia, prior stroke, atrial fibrillation, baseline medical therapy (angiotensin-converting enzyme inhibitors, warfarin), chronic rheumatic heart disease, tricuspid annuloplasty, pulmonary artery systolic pressure, and STS Score ($P<0.05$ on univariate analysis).

Abbreviations: CI, confidence interval; EuroSCORE II, European System for Cardiac Operative Risk Evaluation II; HR, hazard ratio; MELD-XI, Model for End-Stage Liver Disease excluding international normalized ratio; STS score, Society of Thoracic Surgeons Predicted Risk of Mortality Score.

Table S5D. Competing Risk Analysis of baseline hepatorenal function (MELD-XI) for predicting cardiovascular death and heart failure hospitalization

	**Unadjusted**	**EuroSCORE II model***	**STS Score model†**
	SHR (95% CI)	SHR (95% CI)	SHR (95% CI)
**Hepatorenal function**			
MELD-XI, continuous	1.08 (1.06-1.11)	1.06 (1.02-1.10)	1.06 (1.02-1.10)
MELD-XI, categorical			
Normal hepatorenal function	1.00	1.00	1.00
Hepatorenal dysfunction (MELD-XI >12.43)	2.78 (2.02-3.82)	1.65 (1.11-2.45)	1.71 (1.12-2.61)

*Adjusted for atrial fibrillation, heart failure, significant tricuspid regurgitation, pulmonary artery systolic pressure, and EuroSCORE II (P<0.05 on univariate analysis).

†Adjusted for atrial fibrillation, heart failure, pulmonary artery systolic pressure, and STS Score (P<0.05 on univariate analysis).

Abbreviations: CI, confidence interval; CONUT, EuroSCORE II, European System for Cardiac Operative Risk Evaluation II; MELD-XI, Model for End-Stage Liver Disease excluding international normalized ratio; SHR, subdistribution hazard ratio; STS score, Society of Thoracic Surgeons Predicted Risk of Mortality Score.

Table S5E. Competing Risk Analysis of baseline hepatorenal function (MELD-XI) for predicting cardiovascular death

	**Unadjusted**	**EuroSCORE II model***	**STS Score model†**
	SHR (95% CI)	SHR (95% CI)	SHR (95% CI)
**Hepatorenal function**			
MELD-XI, continuous	1.12 (1.09-1.16)	1.10 (1.06-1.14)	1.08 (1.03-1.14)
MELD-XI, categorical			
Normal hepatorenal function	1.00	1.00	1.00
Hepatorenal dysfunction (MELD-XI >12.43)	4.53 (2.64-7.76)	3.23 (1.83-5.70)	3.18 (1.71-5.92)

*Adjusted for hypertension, significant tricuspid regurgitation, and EuroSCORE II (P<0.05 on univariate analysis).

†Adjusted for hypertension and STS Score (P<0.05 on univariate analysis).

Abbreviations: CI, confidence interval; EuroSCORE II, European System for Cardiac Operative Risk Evaluation II; MELD-XI, Model for End-Stage Liver Disease excluding international normalized ratio; SHR, subdistribution hazard ratio; STS score, Society of Thoracic Surgeons Predicted Risk of Mortality Score.

Table S6A. Baseline characteristics of the study population according to nutritional status (CONUT)

Characteristics	Overall (n=909)	Normal nutritional status (n=355)	Mild malnutrition (n=480)	Moderate to severe malnutrition (n=74)	P value
**Demographic and anthropometric characteristics**					
Age, years	63 (57-69)	60 (54-66)†‡	65 (59-70)*	64 (57-71)*	<0.001
Male	431 (47.4)	161 (45.4)	232 (48.3)	38 (51.4)	0.54
Height, cm	159 (153-166)	160 (155-167)	159 (153-165)	158 (153-165)	0.29
Weight, kg	58 (50-67)	61 (53-69)†‡	57 (50-66)*	53 (46-63)*	<0.001
Body mass index, kg/m^2	23 (20.6-25.6)	24 (21-26)†‡	23 (20-26)*	21 (19-25)*	<0.001
NYHA Class III/IV	69 (7.6)	20 (5.6)‡	38 (7.9)	11 (14.9)*	0.022
**Cardiovascular risk factors and cardiovascular disease**					
Hypertension	290 (31.9)	93 (26.2)†	175 (36.5)*	22 (29.7)	0.01
Diabetes Mellitus	165 (18.2)	33 (9.3)†‡	110 (22.9)*	22 (29.7)*	<0.001
Dyslipidemia	231 (25.4)	77 (21.7)	136 (28.3)	18 (24.3)	0.09
Smoking	178 (19.6)	78 (22.0)	86 (17.9)	14 (18.9)	0.34
Prior myocardial infarction	37 (4.1)	9 (2.5)	25 (5.2)	3 (4.1)	0.16
Prior stroke	82 (9.1)	20 (5.6)‡	47 (9.9)‡	15 (20.3)*†	<0.001
Heart failure	393 (43.2)	139 (39.2)‡	207 (43.1)	47 (63.5)*	<0.001
Atrial fibrillation	487 (53.6)	156 (43.9)†‡	280 (58.3)*	51 (68.9)*	<0.001
**Comorbidities**					

Chronic obstructive pulmonary disease	52 (5.7)	15 (4.2)	32 (6.7)	5 (6.8)	0.30
Cancer	52 (5.7)	22 (6.2)	28 (5.8)	2 (2.7)	0.49
**Laboratory examination**					
Hemoglobin, g/dL	12.9 (11.6-14)	13.4 (12.5-14.5)†‡	12.7 (11.5-13.7)*‡	10.6 (9.6-11.8)*†	<0.001
White cell count, x 10^9/L	5.9 (4.9-7.0)	6.1 (5.3-7.0)†‡	5.8 (4.7-7.0)*‡	4.7 (3.8-6.3)*†	<0.001
Platelet count, x 10^9/L	189 (157-227)	201 (174-236)†‡	182 (147-219)*‡	168 (114-203)*†	<0.001
Creatinine, mg/dL	0.9 (0.8-1.1)	0.9 (0.8-1.0)†‡	0.9 (0.8-1.2)*‡	1.2 (0.9-1.5)*†	<0.001
eGFR, ml/min/1.73m^2	75.9 (60.9-90.1)	80.8 (69.2-94.0)†‡	74.0 (59.3-89.2)*‡	60.3 (38.0-75.0)*†	<0.001
AST, U/L	27 (22-35)	26 (22-32)†‡	28 (23-36)*	29 (24-43)*	<0.001
ALT, U/L	21 (16-29)	22 (17-30)	20 (16-28)	19.5 (14-27)	0.03
ALP, U/L	71 (58-90)	67 (55-84)†‡	73 (58-90.2)*‡	98 (68.2-134)*†	<0.001
Total bilirubin, mg/dL	0.7 (0.5-1.1)	0.6 (0.5-0.9)†‡	0.8 (0.6-1.2)*‡	1.2 (0.9-2.1)*†	<0.001
Total cholesterol, mg/dL	159 (134-188)	189 (166-210)†‡	143 (125-164)*‡	127 (113-139)*†	<0.001
Albumin, g/dL	4.2 (4-4.4)	4.3 (4-4.5)†‡	4.2 (4-4.4)*‡	3.7 (3.3-4)*†	<0.001
**Valvular heart disease and echocardiographic variables**					
MS ≥ moderate	229 (25.2)	89 (28.8)	129 (31.9)	11 (17.7)	0.07
MR ≥ moderate	411 (45.2)	174 (49.6)	207 (43.7)	30 (41.1)	0.17
AS ≥ moderate	322 (35.4)	121 (38.1)	180 (41.2)	21 (31.3)	0.27
AR ≥ moderate	231 (25.4)	96 (28.7)	122 (26.0)	13 (18.3)	0.19
TR ≥ moderate	365 (40.2)	91 (25.6)†‡	222 (46.3)*‡	52 (70.3)*†	<0.001

Chronic Rheumatic Heart Disease	259 (28.5)	91 (25.6)	151 (31.5)	17 (23.0)	0.14
LV Mass, g	224 (176-294)	224 (175-291)	225 (175-290)	224 (194-297)	0.38
LVEF, %	60 (55-60)	60 (55-60)‡	60 (55-60)	57.5 (46.2-60)*	0.01
Preserved, ≥50%	763 (84.3)	305 (86.4)‡	403 (84.3)	55 (74.3)*	0.03
Mid-range, 40% - 49%	57 (6.3)	20 (5.7)	33 (6.9)	4 (5.4)	0.73
Reduced, <40%	55 (6.1)	17 (4.8)‡	27 (5.6)‡	11 (14.9)*	0.004
PASP, mmHg	40 (35-50)	40 (30-46)†‡	45 (35-53)*	45 (40-55)*	<0.001
**Medications**					
ACEI	286 (31.5)	97 (27.3)	164 (34.2)	25 (33.8)	0.10
ARB	142 (15.6)	54 (15.2)	80 (16.7)	8 (10.8)	0.42
Aldactone	117 (12.9)	24 (6.8)†‡	68 (14.2)*‡	25 (33.8)*†	<0.001
Beta blockers	374 (41.1)	139 (39.2)	203 (42.3)	32 (43.2)	0.61
Calcium channel blockers	185 (20.4)	68 (19.2)	105 (21.9)	12 (16.2)	0.41
Digoxin	269 (29.6)	90 (25.4)‡	146 (30.4)‡	33 (44.6)*†	0.004
Statin	373 (41)	103 (29.0)†	245 (51.0)*‡	25 (33.8)†	<0.001
Warfarin	417 (45.9)	128 (36.1)†‡	239 (49.8)*‡	50 (67.6)*†	<0.001
**Cardiac surgery risk-stratification models**					
EuroScore II	2.4 (1.3-4.5)	1.8 (1.0-3.0)†‡	2.8 (1.7-5.5)*‡	5.9 (3.2-9.5)*†	<0.001
STS Score	1.5 (0.9-2.8)	1.1 (0.6-1.9)†‡	1.8 (1.1-3.2)*‡	3.3 (1.7-5.5)*†	<0.001
**Valvular surgery details**					

Aortic valve replacement	460 (50.7)	179 (50.4)	252 (52.5)	29 (39.7)	0.13
Mitral valve procedure	554 (61)	219 (61.9)	292 (60.8)	43 (58.1)	0.83
Mitral valve replacement	295 (32.5)	106 (29.9)	168 (35.0)	21 (28.4)	0.22
Mitral valve repair	259 (28.6)	113 (31.9)	124 (25.9)	22 (29.7)	0.16
Tricuspid annuloplasty	319 (35.2)	90 (25.4)†‡	189 (39.5)*‡	40 (54.8)*†	<0.001
Concomitant CABG	107 (11.8)	24 (6.8)†‡	70 (14.6)*	13 (17.8)*	0.001

Values are expressed as median (interquartile range) or number (percentage). P value by Kruskal-Wallis H test for non-normally distributed continuous variables. P value by χ^2 test for categorical variables (Bonferroni correction: *P<0.05 vs normal nutritional status, †P<0.05 vs mild malnutrition [CONUT score of 2 to 4], ‡P<0.05 vs moderate to severe malnutrition [CONUT score of 5-12]).

Abbreviations: ACEI, angiotensin-converting enzyme inhibitors; ARB, angiotensin II receptor blockers; ALP, alkaline phosphatase; ALT, alanine aminotransaminase; AR, aortic regurgitation; AS, aortic stenosis; AST, aspartate aminotransferase; CABG, coronary artery bypass grafting; eGFR, estimated glomerular filtration rate; EuroSCORE II, European System for Cardiac Operative Risk Evaluation II; LVEF; left ventricular ejection fraction; PASP, pulmonary artery systolic pressure; MR, mitral regurgitation; MS, mitral stenosis; NYHA, New York Heart Association; STS score, Society of Thoracic Surgeons Predicted Risk of Mortality Score; TR, tricuspid regurgitation.

Table S6B. Univariate and multivariate logistic regression models showing predictors of baseline malnutrition (CONUT) in patients undergoing valvular surgery

	**Univariate analysis**		**Multivariate analysis (EuroSCORE II model)**		**Multivariate analysis (STS Score model)**	
	OR (95% CI)	P value	OR (95% CI)	P value	OR (95% CI)	P value
**Demographic and anthropometric characteristics**						
Age, years	1.06 (1.04-1.07)	<0.001				
Male	1.15 (0.88-1.50)	0.32				
Body mass index, kg/m^2	0.94 (0.91-0.98)	0.002	0.93 (0.89-0.98)	0.010	0.94 (0.89-0.99)	0.021
NYHA functional class III/IV	1.63 (0.96-2.84)	0.08				
**Cardiovascular risk factors and cardiovascular disease**						
Hypertension	1.55 (1.16-2.09)	0.003	1.92 (1.20-3.13)	0.007		
Diabetes Mellitus	3.05 (2.05-4.65)	<0.001				
Smoking	0.78 (0.56-1.09)	0.147				
Dyslipidemia	1.39 (1.02-1.91)	0.040	1.00 (0.62-1.62)	0.994	1.13 (0.71-1.81)	0.609
Prior myocardial infarction	2.05 (0.99-4.65)	0.066				
Prior stroke	2.13 (1.28-3.67)	0.005	1.70 (0.86-3.53)	0.137	1.77 (0.85-3.89)	0.138
Atrial fibrillation	1.89 (1.45-2.48)	<0.001	0.95 (0.61-1.48)	0.827	0.93 (0.61-1.40)	0.726
Heart failure	1.32 (1.00-1.73)	0.047	0.77 (0.52-1.13)	0.178		

**Valvular heart disease and echocardiographic variables**						
MS ≥ moderate	1.06 (0.77-1.45)	0.725				
MR ≥ moderate	0.78 (0.59-1.02)	0.067				
AS ≥ moderate	1.08 (0.81-1.44)	0.601				
AR ≥ moderate	0.83 (0.61-1.13)	0.233				
TR ≥ moderate	2.85 (2.14-3.82)	<0.001	2.02 (1.30-3.16)	0.002		
LV Mass, g	1.001 (0.999-1.002)	0.449				
LVEF, %	0.98 (0.97-0.99)	0.008				
PASP, mmHg	1.03 (1.02-1.04)	<0.001	1.01 (1.00-1.02)	0.188	1.01 (1.00-1.03)	0.036
**Cardiac surgery risk-stratification models**						
EuroSCORE II	1.14 (1.10-1.19)	<0.001	1.15 (1.07-1.25)	<0.001		
STS Score	1.39 (1.28-1.50)	<0.001			1.34 (1.18-1.56)	<0.001
**Hepatorenal dysfunction**						
eGFR, ml/min/1.73m^2	0.98 (0.97-0.98)	<0.001				
MELD-XI, continuous	1.29 (1.21-1.39)	<0.001	1.23 (1.13-1.37)	<0.001	1.26 (1.14-1.40)	<0.001

Abbreviations: AR, aortic regurgitation; AS, aortic stenosis; CI, confidence interval; CONUT, Controlling Nutritional Status score; EuroSCORE II, European System for Cardiac Operative Risk Evaluation II; LV, left ventricle; LVEF, left ventricular ejection fraction; PASP, pulmonary artery systolic pressure; MELD-XI, Model for End-Stage Liver Disease excluding international normalized ratio; MR, mitral regurgitation; MS, mitral stenosis; NYHA, New York Heart Association; OR, odds ratio; STS score, Society of Thoracic Surgeons Predicted Risk of Mortality Score; TR, tricuspid regurgitation.

Table S6C. Cox proportional hazards analysis of baseline nutritional status (CONUT) for predicting all-cause mortality and adverse events

	Multivariate analysis							
	EuroSCORE II model				STS Score model			
	All-cause mortality*		Adverse events†		All-cause mortality‡		Adverse events§	
	HR (95% CI)	P value	HR (95% CI)	P value	HR (95% CI)	P value	HR (95% CI)	P value
**Nutritional status**								
CONUT, continuous	1.41 (1.25-1.58)	<0.001	1.28 (1.16-1.42)	<0.001	1.43 (1.27-1.62)	<0.001	1.30 (1.17-1.45)	<0.001
CONUT, categorical								
Normal nutrition	1.00		1.00		1.00		1.00	
Mild malnutrition	3.81 (1.87-7.75)	<0.001	2.06 (1.29-3.28)	0.002	3.88 (1.89-7.93)	<0.001	1.99 (1.24-3.20)	0.004
Moderate to severe malnutrition	7.97 (3.58-17.77)	<0.001	4.17 (2.35-7.41)	<0.001	7.95 (3.42-18.48)	<0.001	3.89 (2.14-7.10)	<0.001

*Adjusted for hypertension, dyslipidemia, prior stroke, baseline warfarin therapy, type of valvular lesion, type of valvular surgery, EuroSCORE II (P<0.05 on univariate analysis).

†Adjusted for hypertension, dyslipidemia, prior stroke, atrial fibrillation, heart failure, baseline medical therapy (angiotensin-converting enzyme inhibitors, warfarin), chronic rheumatic heart disease, type of valvular lesion, tricuspid annuloplasty, pulmonary artery systolic pressure and EuroSCORE II (P<0.05 on univariate analysis).

‡Adjusted for dyslipidemia, prior stroke, baseline warfarin therapy, type of valvular surgery, and STS Score ($P<0.05$ on univariate analysis).

§Adjusted for dyslipidemia, prior stroke, atrial fibrillation, baseline medical therapy (angiotensin-converting enzyme inhibitors, warfarin), chronic rheumatic heart disease, tricuspid annuloplasty, pulmonary artery systolic pressure, and STS Score ($P<0.05$ on univariate analysis).

Abbreviations: CI, confidence interval; CONUT, Controlling Nutritional Status score; EuroSCORE II, European System for Cardiac Operative Risk Evaluation II; HR, hazard ratio; STS score, Society of Thoracic Surgeons Predicted Risk of Mortality Score.

Table S6D. Competing Risk Analysis of baseline nutritional status (CONUT) for predicting heart failure hospitalization

	**Unadjusted**	**EuroSCORE II model***	**STS Score model†**
	SHR (95% CI)	SHR (95% CI)	SHR (95% CI)
**Nutritional Status**			
CONUT, continuous	1.44 (1.32-1.56)	1.29 (1.16-1.42)	1.26 (1.12-1.41)
CONUT, categorical			
Normal nutrition	1.00	1.00	1.00
Mild malnutrition	2.46 (1.53-3.95)	2.09 (1.30-3.37)	1.96 (1.18-3.24)
Moderate to severe malnutrition	5.92 (3.39-10.36)	4.00 (2.23-7.17)	3.25 (1.64-6.43)

*Adjusted for atrial fibrillation, heart failure, significant tricuspid regurgitation, pulmonary artery systolic pressure, and EuroSCORE II (P<0.05 on univariate analysis).

†Adjusted for atrial fibrillation, heart failure, pulmonary artery systolic pressure, and STS Score (P<0.05 on univariate analysis).

Abbreviations: CI, confidence interval; CONUT, Controlling Nutritional Status score; EuroSCORE II, European System for Cardiac Operative Risk Evaluation II; MELD-XI, Model for End-Stage Liver Disease excluding international normalized ratio; SHR, subdistribution hazard ratio; STS score, Society of Thoracic Surgeons Predicted Risk of Mortality Score.

Table S6E. Competing Risk Analysis of baseline nutritional status (CONUT) for predicting cardiovascular death

	**Unadjusted**	**EuroSCORE II model***	**STS Score model†**
	SHR (95% CI)	SHR (95% CI)	SHR (95% CI)
**Nutritional Status**			
CONUT, continuous	1.62 (1.44-1.83)	1.53 (1.35-1.74)	1.61 (1.41-1.84)
CONUT, categorical			
Normal nutrition	1.00	1.00	1.00
Mild malnutrition	5.21 (2.03-13.4)	3.94 (1.55-10.02)	3.97 (1.50-10.49)
Moderate to severe malnutrition	16.42 (5.98-45.1)	10.31 (3.73-28.51)	11.21 (3.78-33.26)

*Adjusted for hypertension, significant tricuspid regurgitation, and EuroSCORE II (P<0.05 on univariate analysis).

†Adjusted for hypertension and STS Score (P<0.05 on univariate analysis).

Abbreviations: CI, confidence interval; CONUT, Controlling Nutritional Status score; EuroSCORE II, European System for Cardiac Operative Risk Evaluation II; MELD-XI, Model for End-Stage Liver Disease excluding international normalized ratio; SHR, subdistribution hazard ratio; STS score, Society of Thoracic Surgeons Predicted Risk of Mortality Score.

Table S7. Calibration of the Cox proportional hazards models for predicting all-cause mortality and adverse events

Outcome	**EuroSCORE II model**		**STS Score model**	
	Wald Chi-square	P value	Wald Chi-square	P value
All-cause mortality	2.22	0.137	1.93	0.165
Adverse events	4.90	0.180	1.36	0.506

Abbreviations: EuroSCORE II, European System for Cardiac Operative Risk Evaluation II; STS score, Society of Thoracic Surgeons Predicted Risk of Mortality Score.

Table S8. Incremental prognostic and discriminatory value of adding serum albumin to EuroSCORE II and STS Score

**Models**	**C-statistics**	**P value**	**cNRI**	**P value**	**IDI**	**P value**
**Death**						
EuroSCORE II	0.73	<0.001	Ref		Ref	
EuroSCORE II + Albumin	0.72	0.602	0.21	0.053	0.01	0.088
STS Score	0.72	<0.001	Ref		Ref	
STS Score + Albumin	0.74	0.209	0.25	0.027	0.01	0.032

Abbreviations: cNRI, continuous net reclassification improvement; EuroSCORE II, European System for Cardiac Operative Risk Evaluation II; IDI, integrated discrimination improvement; STS score, Society of Thoracic Surgeons Predicted Risk of Mortality Score.

Table S9A. Changes in Calibration of the Cox proportional hazards models for predicting all-cause mortality with inclusion of the MELD-XI and CONUT scores

	**EuroSCORE II***	**EuroSCORE II + MELD-XI**	**EuroSCORE II + CONUT**	**EuroSCORE II + MELD-XI + CONUT**	**STS Score†**	**STS Score + MELD-XI**	**STS Score + CONUT**	**STS Score + MELD-XI + CONUT**
Likelihood Ratio Test	-463	-445	-449	-437	-391	-382	-379	-370
P value‡	–	<0.001	<0.001	<0.001	–	<0.001	<0.001	<0.001
AIC	943	911	918	896	794	777	771	755
BIC	965	935	942	922	807	793	786	773

*Fully adjusted model comprising baseline comorbidities (hypertension, dyslipidemia, prior stroke), type of valvular lesion (significant mitral regurgitation, significant tricuspid regurgitation), baseline warfarin therapy, type of valvular procedure (aortic valve replacement and mitral valve procedure), and EuroSCORE II as in Table 2.

†Fully adjusted model comprising baseline comorbidities (dyslipidemia, prior stroke), type of valvular lesion (significant mitral regurgitation, significant tricuspid regurgitation), baseline warfarin therapy, type of valvular procedure (aortic valve replacement and mitral valve procedure), and STS Score as in Table 2.

‡Compared to EuroSCORE II / STS Score model.

Abbreviations: AIC, Akaike information criteria; BIC, Bayesian information criteria; CONUT, Controlling Nutritional Status score; EuroSCORE II, European System for Cardiac Operative Risk Evaluation II; MELD-XI, Model for End-Stage Liver Disease excluding international normalized ratio; STS score, Society of Thoracic Surgeons Predicted Risk of Mortality Score.

Table S9B. Changes in Calibration of the Cox proportional hazards models for predicting adverse events with inclusion of the MELD-XI and CONUT scores

	**EuroSCORE II***	**EuroSCORE II + MELD-XI**	**EuroSCORE II + CONUT**	**EuroSCORE II + MELD-XI + CONUT**	**STS Score†**	**STS Score + MELD-XI**	**STS Score + CONUT**	**STS Score + MELD-XI + CONUT**
Likelihood Ratio Test	-905	-897	-893	-889	-789	-786	-779	-778
P value‡	–	<0.001	<0.001	<0.001	–	0.028	<0.001	<0.001
AIC	1836	1821	1815	1807	1596	1593	1596	1579
BIC	1875	1864	1857	1853	1622	1622	1621	1610

*Fully adjusted model comprising baseline comorbidities (hypertension, dyslipidemia, prior stroke, atrial fibrillation, heart failure), type of valvular lesion (significant mitral regurgitation, significant tricuspid regurgitation), chronic rheumatic heart disease, baseline medical therapy (angiotensin-converting enzyme inhibitors, warfarin), tricuspid annuloplasty and EuroSCORE II as in Table S2D.

†Fully adjusted model comprising baseline comorbidities (dyslipidemia, prior stroke, atrial fibrillation), chronic rheumatic heart disease, baseline medical therapy (angiotensin-converting enzyme inhibitors, warfarin), tricuspid annuloplasty and STS Score as in Table S2D.

‡Compared to EuroSCORE II / STS Score model.

Abbreviations: AIC, Akaike information criteria; BIC, Bayesian information criteria; CONUT, Controlling Nutritional Status score; EuroSCORE II, European System for Cardiac Operative Risk Evaluation II; MELD-XI, Model for End-Stage Liver Disease excluding international normalized ratio; STS score, Society of Thoracic Surgeons Predicted Risk of Mortality Score.

Table S10. Baseline characteristics of the secondary cohort compared with the rest of the study population

Characteristics	Patients included in the secondary cohort (n=707)	Patients not included in the secondary cohort (n=202)	P value
**Demographic and anthropometric characteristics**			
Age, years	63 (57-69)	62 (55-69)	0.221
Male	339 (47.9)	92 (45.5)	0.576
Height, cm	160 (153-166)	158 (153-166)	0.257
Weight, kg	59 (51-67)	56 (50-66)	0.052
Body mass index, kg/m^2	23.4 (20.6-25.9)	22.1 (20.3-24.8)	0.010
NYHA Class III/IV	51 (7.2)	18 (8.9)	0.451
**Cardiovascular risk factors and cardiovascular disease**			
Hypertension	236 (33.4)	54 (26.7)	0.087
Diabetes Mellitus	136 (19.2)	29 (14.4)	0.121
Dyslipidemia	190 (26.9)	41 (20.3)	0.067
Smoking	138 (19.5)	40 (19.8)	1.000
Prior myocardial infarction	29 (4.1)	8 (4.0)	1.000
Prior stroke	62 (8.8)	20 (10.0)	0.676
Heart failure	321 (45.4)	72 (35.6)	0.016
Atrial fibrillation	377 (53.3)	110 (54.4)	0.811
**Comorbidities**			

Chronic obstructive pulmonary disease	38 (5.4)	14 (6.9)	0.492
Cancer	34 (4.8)	18 (8.9)	0.038
**Laboratory examination**			
Hemoglobin, g/dL	12.9 (11.8-14.1)	12.5 (11.0-13.7)	0.005
White cell count, x 10^9/L	5.9 (4.8-7.1)	5.9 (4.9-6.7)	0.551
Platelet count, x 10^9/L	189 (159-226)	188 (152-234)	0.942
Creatinine, mg/dL	0.93 (0.78-1.12)	0.89 (0.77-1.21)	0.833
eGFR, ml/min/1.73m^2	75.5 (61.5-89.5)	78.3 (57.2-93.7)	0.687
AST, U/L	27 (23-34)	28 (22-37)	0.655
ALT, U/L	21 (16-29)	20 (16-28)	0.436
ALP, U/L	71 (58-89)	73 (59-100)	0.039
Total bilirubin, mg/dL	0.75 (0.53-1.10)	0.73 (0.53-1.23)	0.345
Total cholesterol, mg/dL	160 (134-189)	154 (132-185)	0.341
Albumin, g/dL	4.2 (4-4.4)	4.2 (3.9-4.4)	0.463
**Valvular heart disease and echocardiographic variables**			
MS ≥ moderate	176 (29.6)	53 (29.3)	1.000
MR ≥ moderate	325 (46.6)	86 (43.0)	0.377
AS ≥ moderate	255 (40.3)	67 (35.4)	0.236
AR ≥ moderate	184 (27.2)	47 (23.6)	0.317
TR ≥ moderate	272 (38.5)	93 (46.0)	0.061

Chronic Rheumatic Heart Disease	197 (27.9)	62 (30.7)	0.486
LV Mass, g	225 (176-294)	218 (176-293)	0.444
LVEF, %	60 (55-60)	60 (51-60)	0.949
Preserved, ≥50%	591 (84.1)	172 (85.1)	0.743
Mid-range, 40% - 49%	45 (6.4)	12 (5.9)	0.871
Reduced, <40%	43 (6.1)	12 (5.9)	1.000
PASP, mmHg	40 (35-50)	45 (35-51)	0.125
**Medications**			
ACEI	227 (32.1)	59 (29.2)	0.441
ARB	110 (15.6)	32 (15.8)	1.000
Aldactone	80 (11.3)	37 (18.3)	0.012
Beta blockers	290 (41.0)	84 (41.6)	0.935
Calcium channel blockers	146 (20.7)	39 (19.3)	0.694
Digoxin	209 (29.6)	60 (29.7)	1.000
Statin	304 (43.0)	69 (34.2)	0.028
Warfarin	322 (45.5)	95 (47.0)	0.749
**Cardiac surgery risk-stratification models**			
EuroScore II	2.43 (1.38-4.39)	2.41 (1.20-4.80)	0.955
STS Score	1.49 (0.88-2.60)	1.58 (0.77-3.48)	0.584
**Valvular surgery details**			

Aortic valve replacement	363 (51.4)	97 (48.0)	0.425
Mitral valve procedure	434 (61.5)	120 (59.4)	0.624
Mitral valve replacement	227 (32.1)	68 (33.7)	0.733
Mitral valve repair	207 (29.4)	52 (25.7)	0.332
Tricuspid annuloplasty	242 (34.3)	77 (38.1)	0.358
Concomitant CABG	92 (13.0)	15 (7.5)	0.035

Values are expressed as median (interquartile range) or number (percentage). P value by Mann-Whitney U test for non-normally distributed continuous variables; P value by χ^2 test for categorical variables.

Abbreviations: ACEI, angiotensin-converting enzyme inhibitors; ARB, angiotensin II receptor blockers; ALP, alkaline phosphatase; ALT, alanine aminotransaminase; AR, aortic regurgitation; AS, aortic stenosis; AST, aspartate aminotransferase; CABG, coronary artery bypass grafting; eGFR, estimated glomerular filtration rate; EuroSCORE II, European System for Cardiac Operative Risk Evaluation II; LVEF; left ventricular ejection fraction; PASP, pulmonary artery systolic pressure; MR, mitral regurgitation; MS, mitral stenosis; NYHA, New York Heart Association; STS score, Society of Thoracic Surgeons Predicted Risk of Mortality Score; TR, tricuspid regurgitation.

Table S11A. Univariate and multivariate linear regression models showing predictors of 1-year change in MELD-XI score (ΔMELD-XI) in patients undergoing valvular surgery

	**Univariate analysis**		**Multivariate analysis**	
	OR (95% CI)	P value	OR (95% CI)	P value
**Demographic and anthropometric characteristics**				
Age, years	1.01 (0.99-1.03)	0.188		
Male	0.76 (0.56-1.03)	0.079		
Body mass index, kg/m^2	1.03 (0.99-1.07)	0.199		
NYHA functional class III/IV	0.39 (0.22-0.69)	0.001	0.54 (0.30-0.99)	0.045
**Cardiovascular risk factors and cardiovascular disease**				
Hypertension	1.58 (1.14-2.20)	0.006	1.23 (0.86-1.74)	0.253
Diabetes Mellitus	1.81 (1.21-2.70)	0.004	1.37 (0.90-2.07)	0.137
Smoking	0.80 (0.54-1.17)	0.249		
Dyslipidemia	1.29 (0.90-1.84)	0.163		
Prior myocardial infarction	2.13 (0.97-4.68)	0.060		
Prior stroke	0.70 (0.41-1.20)	0.195		
Atrial fibrillation	0.61 (0.45-0.83)	0.002	0.74 (0.51-1.07)	0.111
Heart failure	0.67 (0.49-0.91)	0.010	0.78 (0.56-1.10)	0.157
**Valvular heart disease and echocardiographic variables**				

MS ≥ moderate	0.98 (0.69-1.41)	0.932		
MR ≥ moderate	0.58 (0.43-0.79)	<0.001	0.76 (0.52-1.09)	0.134
AS ≥ moderate	1.62 (1.17-2.23)	0.004	1.24 (0.85-1.81)	0.258
AR ≥ moderate	1.34 (0.93-1.91)	0.112		
TR ≥ moderate	0.56 (0.41-0.77)	<0.001	0.97 (0.66-1.42)	0.871
LV Mass, g	1.000 (0.998-1.002)	0.928		
LVEF, %	1.01 (1.00-1.03)	0.125		
PASP, mmHg	0.99 (0.98-1.00)	0.120		
EuroSCORE II	1.01 (0.98-1.05)	0.479		
STS Score	0.99 (0.91-1.07)	0.790		
**Nutritional status**				
Baseline CONUT, continuous	0.85 (0.77-0.95)	0.002		
ΔCONUT	0.89 (0.78-1.01)	<0.001	1.22 (1.00-1.37)	<0.001

Abbreviations: ACEI, angiotensin-converting enzyme inhibitors; ARB, angiotensin II receptor blockers; ALP, alkaline phosphatase; ALT, alanine aminotransaminase; AR, aortic regurgitation; AS, aortic stenosis; AST, aspartate aminotransferase; CABG, coronary artery bypass grafting; CONUT, Controlling Nutritional Status score; ΔCONUT, 1-year change in CONUT score; eGFR, estimated glomerular filtration rate; EuroSCORE II, European System for Cardiac Operative Risk Evaluation II; LVEF; left ventricular ejection fraction; PASP, pulmonary artery systolic pressure; MR, mitral regurgitation; MS, mitral stenosis; NYHA, New York Heart Association; STS score, Society of Thoracic Surgeons Predicted Risk of Mortality Score; TR, tricuspid regurgitation.

Table S11B. Univariate and multivariate linear regression models showing predictors of 1-year change in CONUT score (ΔCONUT) in patients undergoing valvular surgery

	**Univariate analysis**		**Multivariate analysis**	
	OR (95% CI)	P value	OR (95% CI)	P value
**Demographic and anthropometric characteristics**				
Age, years	0.99 (0.98-1.00)	0.182		
Male	1.12 (0.91-1.38)	0.270		
Body mass index, kg/m^2	1.02 (0.99-1.05)	0.138		
NYHA functional class III/IV	0.80 (0.54-1.19)	0.263		
**Cardiovascular risk factors and cardiovascular disease**				
Hypertension	1.00 (0.80-1.24)	0.974		
Diabetes Mellitus	1.09 (0.84-1.41)	0.515		
Smoking	1.23 (0.95-1.59)	0.114		
Dyslipidemia	0.92 (0.73-1.16)	0.491		
Prior myocardial infarction	1.50 (0.90-2.51)	0.124		
Prior stroke	0.83 (0.58-1.20)	0.319		
Atrial fibrillation	0.91 (0.74-1.11)	0.343		
Heart failure	0.91 (0.74-1.11)	0.344		
**Valvular heart disease and echocardiographic variables**				

MS ≥ moderate	1.06 (0.83-1.35)	0.627		
MR ≥ moderate	1.12 (0.91-1.38)	0.269		
AS ≥ moderate	1.04 (0.84-1.30)	0.709		
AR ≥ moderate	0.79 (0.62-1.00)	0.049	0.74 (0.57-0.95)	0.019
TR ≥ moderate	0.73 (0.60-0.91)	0.004	0.85 (0.67-1.083)	0.188
LV Mass, g	1.000 (0.998-1.001)	0.480		
LVEF, %	1.01 (1.00-1.02)	0.020		
PASP, mmHg	0.99 (0.98-1.00)	0.008	0.99 (0.99-1.00)	0.103
EuroSCORE II	0.97 (0.95-0.99)	0.009	0.98 (0.95-1.00)	0.041
STS Score	0.97 (0.92-1.03)	0.292		
**Hepatorenal function**				
Baseline MELD-XI, continuous	0.99 (0.96-1.03)	0.626		
ΔMELD-XI	1.12 (1.07-1.17)	<0.001	1.16 (1.11-1.21)	<0.001

Abbreviations: AR, aortic regurgitation; AS, aortic stenosis; CI, confidence interval; CONUT, Controlling Nutritional Status score; EuroSCORE II, European System for Cardiac Operative Risk Evaluation II; LV, left ventricle; LVEF, left ventricular ejection fraction; PASP, pulmonary artery systolic pressure; MELD-XI, Model for End-Stage Liver Disease excluding international normalized ratio; ΔMELD-XI, 1-year change in MELD-XI score; MR, mitral regurgitation; MS, mitral stenosis; NYHA, New York Heart Association; OR, odds ratio; STS score, Society of Thoracic Surgeons Predicted Risk of Mortality Score; TR, tricuspid regurgitation.

Table S11C. Univariate and multivariate logistic regression models showing predictors of postoperative hepatorenal dysfunction (MELD-XI) and malnutrition (CONUT) in patients undergoing valvular surgery

	**Univariate analysis**		**Multivariate analysis (EuroSCORE II model)**		**Multivariate analysis (STS Score model)**	
	OR (95% CI)	P value	OR (95% CI)	P value	OR (95% CI)	P value
**Demographic and anthropometric characteristics**						
Age, years	1.06 (1.03-1.08)	<0.001				
Male	2.06 (1.36-3.16)	<0.001				
Body mass index, kg/m^2	1.03 (0.97-1.09)	0.300				
NYHA functional class III/IV	1.79 (0.87-3.46)	0.093				
**Cardiovascular risk factors and cardiovascular disease**						
Hypertension	1.75 (1.15-2.65)	0.009	1.87 (1.00-3.51)	0.050		
Diabetes Mellitus	3.42 (2.18-5.33)	<0.001				
Smoking	1.30 (0.78-2.09)	0.302				
Dyslipidemia	1.45 (0.92-2.23)	0.101				
Prior myocardial infarction	2.63 (1.11-5.79)	0.020	0.45 (0.07-2.36)	0.372	0.70 (0.11-3.38)	0.673
Prior stroke	1.38 (0.68-2.60)	0.347				
Atrial fibrillation	1.16 (0.77-1.76)	0.475				
Heart failure	1.94 (1.28-2.95)	0.002	1.12 (0.60-2.10)	0.721		

**Valvular Heart Disease and Echocardiographic variables**						
MS ≥ moderate	0.82 (0.48-1.37)	0.463				
MR ≥ moderate	1.18 (0.78-1.78)	0.441				
AS ≥ moderate	1.05 (0.67-1.62)	0.835				
AR ≥ moderate	0.86 (0.52-1.37)	0.539				
TR ≥ moderate	1.95 (1.29-2.95)	0.002	1.28 (0.65-2.53)	0.481		
LV Mass, g	1.004 (1.002-1.006)	<0.001	1.004 (1.000-1.007)	0.025	1.004 (1.001-1.008)	0.006
LVEF, %	0.97 (0.96-0.99)	0.009				
PASP, mmHg	1.02 (1.01-1.04)	0.005	1.00 (0.98-1.02)	0.663	1.00 (0.98-1.02)	0.984
**Cardiac surgery risk-stratification models**						
EuroSCORE II	1.13 (1.08-1.19)	<0.001	1.14 (1.07-1.23)	<0.001		
STS Score	1.31 (1.19-1.43)	<0.001			1.12 (0.98-1.29)	0.09
**Baseline Hepatorenal function**						
MELD-XI, continuous	1.54 (1.42-1.69)	<0.001	1.47 (1.31-1.65)	<0.001	1.43 (1.28-1.61)	<0.001
**Baseline Nutritional status**						
CONUT, continuous	1.75 (1.53-2.03)	<0.001	1.28 (1.05-1.56)	0.014	1.37 (1.12-1.68)	0.002

Abbreviations: AR, aortic regurgitation; AS, aortic stenosis; CI, confidence interval; CONUT, Controlling Nutritional Status score; EuroSCORE II, European System for Cardiac Operative Risk Evaluation II; LV, left ventricle; LVEF, left ventricular ejection fraction; PASP, pulmonary artery systolic pressure; MELD-XI, Model for End-Stage Liver Disease excluding international normalized ratio; MR, mitral regurgitation; MS, mitral stenosis; NYHA, New York Heart Association; OR, odds ratio; STS score, Society of Thoracic Surgeons Predicted Risk of Mortality Score; TR, tricuspid regurgitation.

Figure S1. Study population flowchart for inclusion of patients undergoing valvular surgery

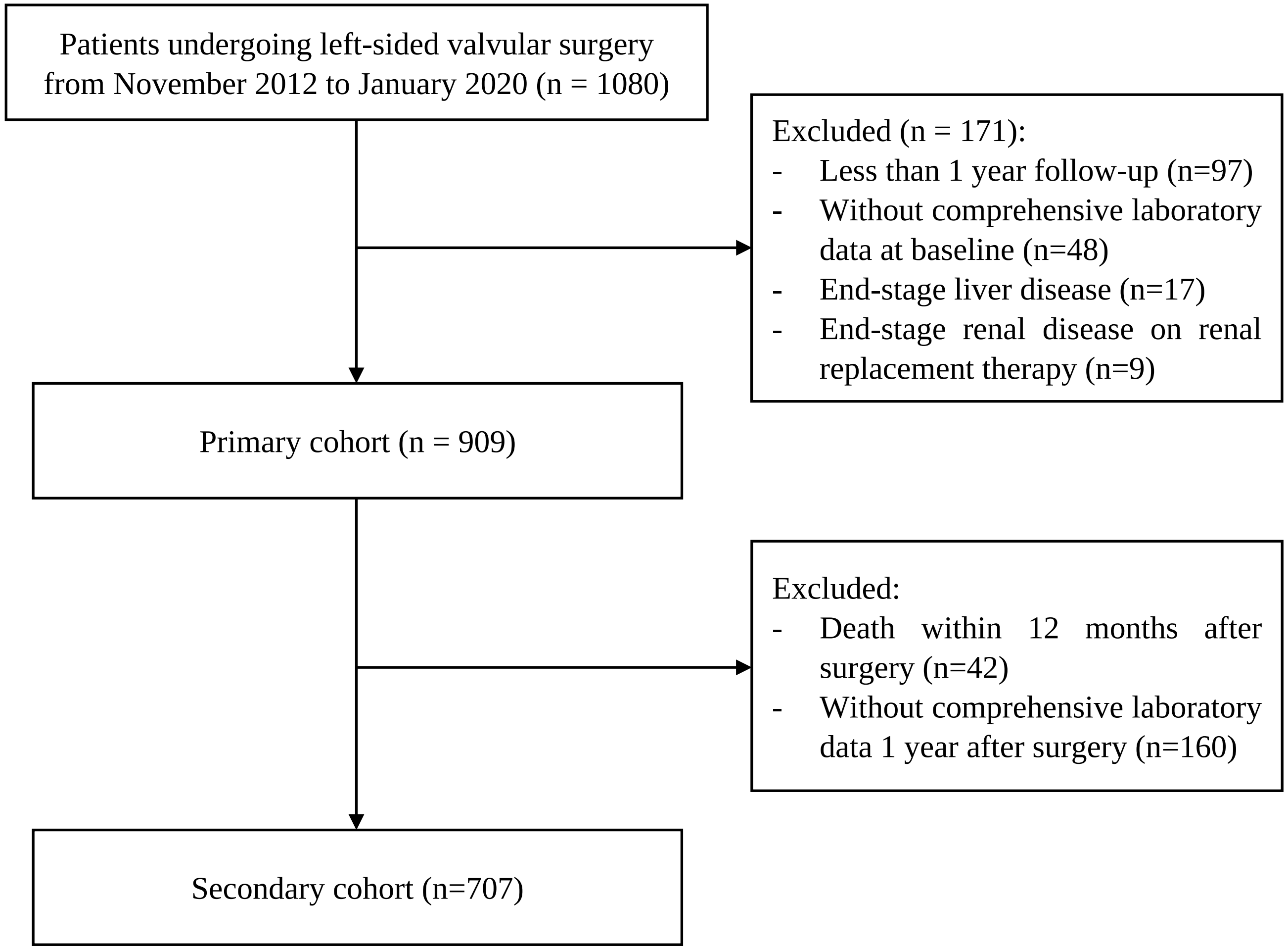

Figure S2. Prevalence and association of hepatorenal dysfunction (MELD-XI) and malnutrition (CONUT) with incidence of all-cause mortality and adverse events

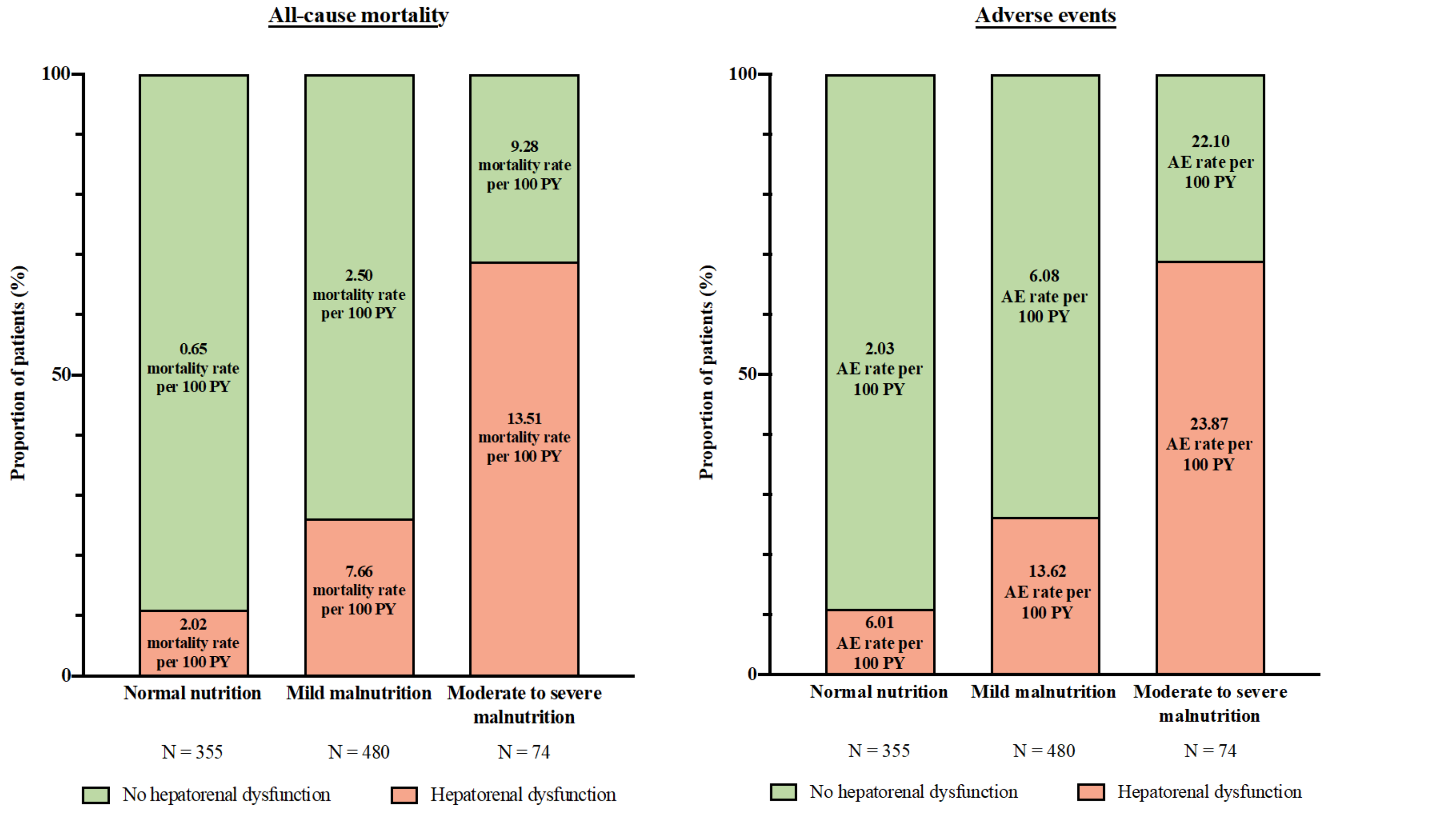

Abbreviations: AE, adverse event; PY, person-years.

Figure S3. Kaplan-Meier curves for all-cause mortality and adverse events by baseline hepatorenal dysfunction (MELD-XI) and malnutrition (CONUT)

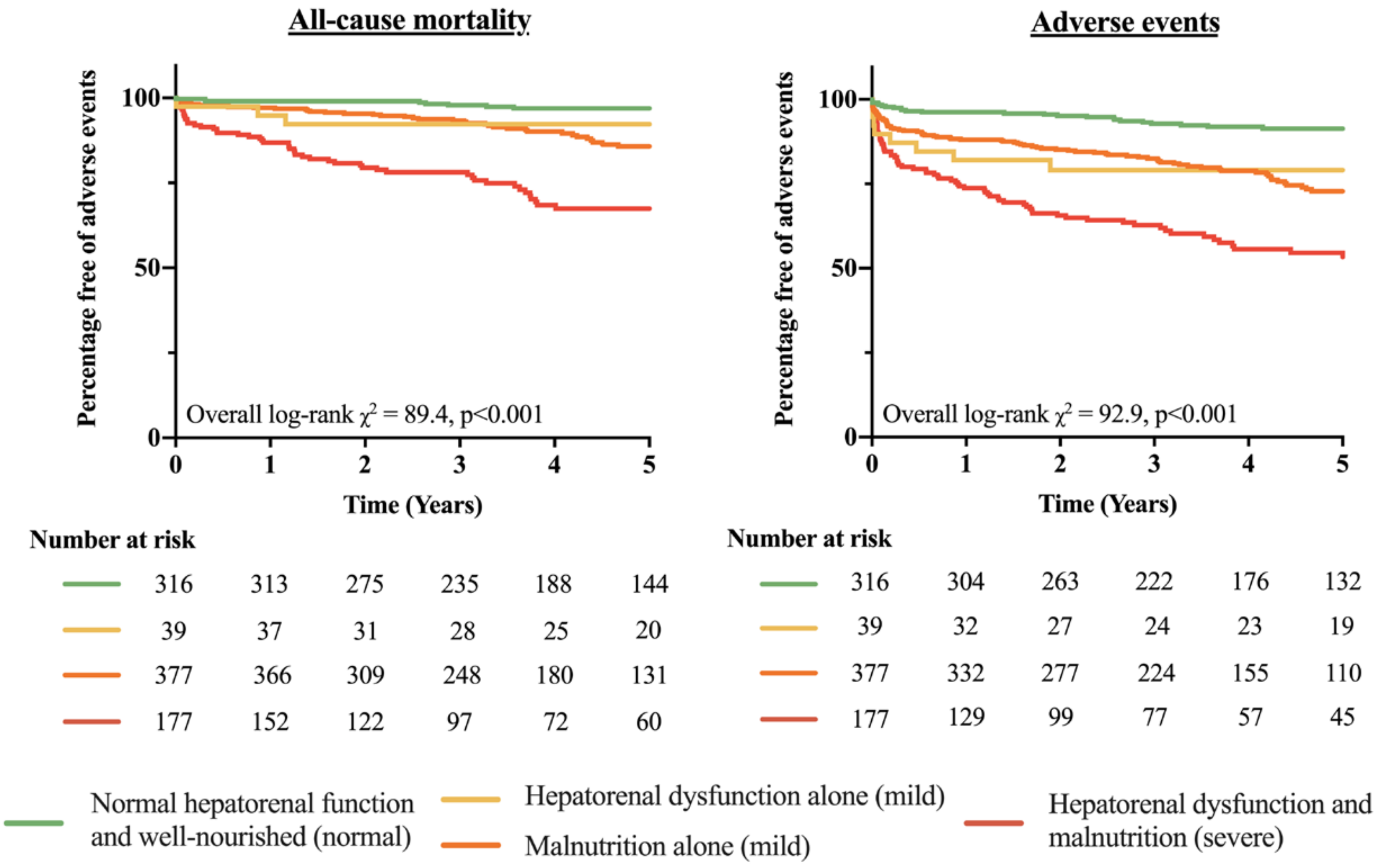

Figure S4. Strength of association of clinical and echocardiographic covariates and risk scores with mortality and adverse outcomes according to the explained log-likelihood (χ^2) for each predictor in patients undergoing valvular surgery

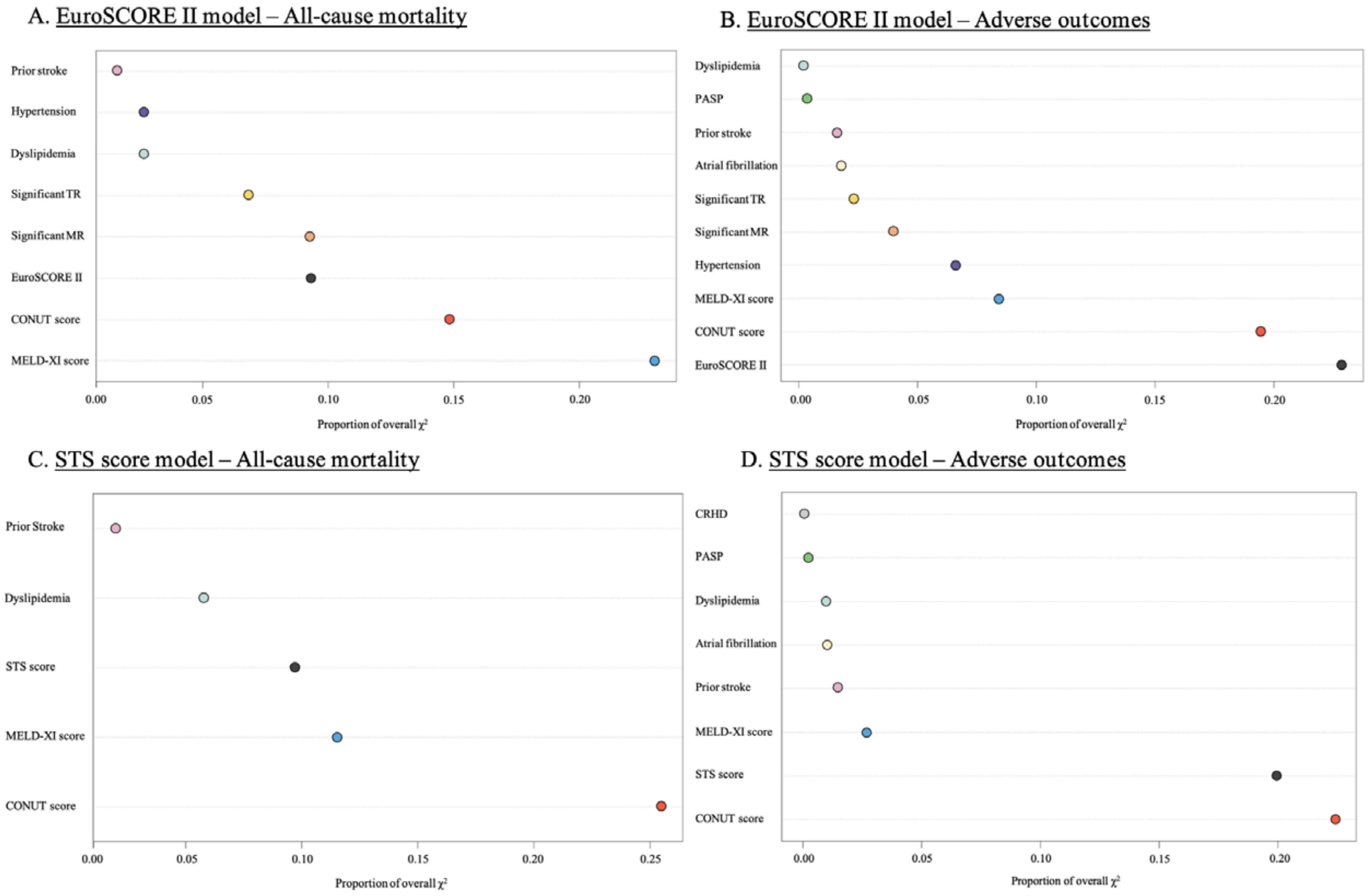

E. Clinical model – All-cause mortality   F. Clinical model – Adverse outcomes

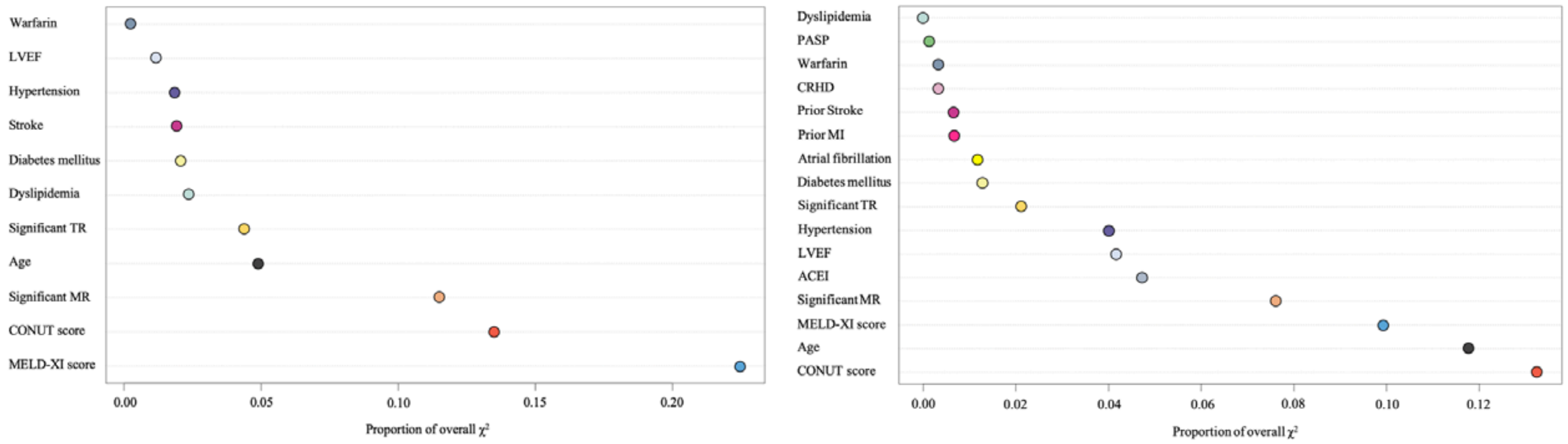

Abbreviations: ACEI, angiotensin-converting enzyme inhibitors; CONUT, Controlling Nutritional Status score; CRHD, chronic rheumatic heart disease; EuroSCORE II, European System for Cardiac Operative Risk Evaluation II; LVEF; left ventricular ejection fraction; PASP, pulmonary artery systolic pressure; MELD-XI, Model for End-Stage Liver Disease excluding international normalized ratio; MI, myocardial infarction; MR, mitral regurgitation; NYHA, New York Heart Association; STS score, Society of Thoracic Surgeons Predicted Risk of Mortality Score; TR, tricuspid regurgitation.
